# Stealth nanorods *via* the aqueous living crystallisation-driven self-assembly of poly(2-oxazoline)s†

**DOI:** 10.1039/d1sc00938a

**Published:** 2021-04-12

**Authors:** John R. Finnegan, Emily H. Pilkington, Karen Alt, Md. Arifur Rahim, Stephen J. Kent, Thomas P. Davis, Kristian Kempe

**Affiliations:** ARC Centre of Excellence in Convergent Bio-Nano Science and Technology, Drug Delivery, Disposition and Dynamics, Monash Institute of Pharmaceutical Sciences, Monash University Parkville Victoria 3052 Australia kristian.kempe@monash.edu; ARC Centre of Excellence in Convergent Bio-Nano Science, Department of Microbiology and Immunology, Peter Doherty Institute for Infection and Immunity, The University of Melbourne Parkville Victoria 3010 Australia; NanoTheranostics Laboratory, Australian Centre for Blood Diseases, Monash University Melbourne Victoria 3004 Australia; School of Chemical Engineering, University of New South Wales (UNSW) Sydney NSW 2052 Australia; Australian Institute for Bioengineering and Nanotechnology, The University of Queensland Brisbane QLD 4072 Australia t.davis@uq.edu.au; Materials Science and Engineering, Monash University Clayton VIC 3800 Australia

## Abstract

The morphology of nanomaterials critically influences their biological interactions. However, there is currently a lack of robust methods for preparing non-spherical particles from biocompatible materials. Here, we combine ‘living’ crystallisation-driven self-assembly (CDSA), a seeded growth method that enables the preparation of rod-like polymer nanoparticles, with poly(2-oxazoline)s (POx), a polymer class that exhibits ‘stealth’ behaviour and excellent biocompatibility. For the first time, the ‘living’ CDSA process was carried out in pure water, resulting in POx nanorods with lengths ranging from ∼60 to 635 nm. *In vitro* and *in vivo* study revealed low immune cell association and encouraging blood circulation times, but little difference in the behaviour of POx nanorods of different length. The stealth behaviour observed highlights the promising potential of POx nanorods as a next generation stealth drug delivery platform.

## Abstract 2

Triggered by heating, a poly(2-alkyl-2-oxazoline) block copolymer undergoes seeded growth in water forming length tuneable nanorods. Morphology and composition combine to impart low immune cell association and promising blood circulation lifetimes.

## Graphical abstract

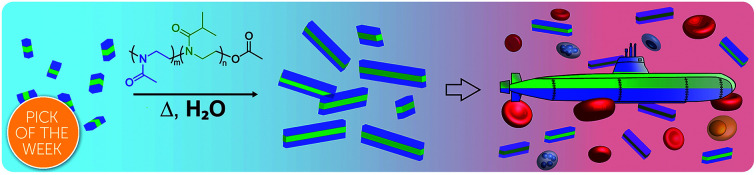

## Introduction

Nanoparticles (NPs) designed to circulate through the blood stream before reaching a specific tissue or organ have the potential to improve the efficacy of current therapeutics.^1^ Polymeric materials feature heavily in the list of FDA approved nanomedicines and drug loaded block copolymer (BCP) micelles have yielded encouraging results in late stage clinical trials.^1,2^ These core–shell structures are prepared *via* solution-phase self-assembly, a process that predominately results in particles with a spherical morphology, and by which the formation of pure phases of non-spherical particles remains a significant challenge.^3–5^ In addition, under conditions where low curvature morphologies such as cylinders and discs are favoured, the particles produced exhibit broad size distributions. Due to these synthetic challenges, the *in vivo* and *in vitro* behaviour of high aspect ratio BCP micelles have been studied to a far lesser extent than that of spherical morphologies. This gap in the literature is made all the more significant by the observation that fibrillar nanomaterials can outperform their spherical analogues as potential nanomedicines showing, for example, increased blood circulation time and superior tumour penetration.^6–10^

BCPs with a crystallisable solvophobic block display significantly different self-assembly behaviour compared to their amorphous analogues. These materials favour the formation of low curvature morphologies and as a consequence, pure suspensions of fibre- or platelet-like NPs can be obtained by crystallisation-driven self-assembly (CDSA).^4,11^ The exact morphology formed under a given set of conditions is determined by the volume ratio of the core- to corona-forming block and whether the process takes place under kinetic or thermodynamic control.^12,13^ Significantly, the Manners and Winnik groups have demonstrated that the size of crystalline-core BCP NPs can be precisely controlled by a seeded growth process termed living CDSA.^14–16^ In the case of fibre-like micelles, length disperse NPs are first formed *via* uncontrolled spontaneous nucleation. These are then fragmented by vigorous ultrasonication resulting in seeds with an average length between 20 and 100 nm, and a narrow length distribution. In the final step, BCP is added as a unimer solution to a suspension of seeds resulting in elongated fibres with well-controlled average lengths directly proportional to the mass ratio of unimer to seeds (*m*_unimer_/*m*_seed_). Alternatively, seed micelle suspensions can be heated to a range of temperatures such that an increasing percentage of the seeds are dissolved to generate unimer. Upon cooling, this unimer will add to the remaining seed micelles resulting in a suspension of well-defined nanofibres with lengths proportional to the amount of unimer generated/seeds remaining during annealing. This process is termed self-seeding.^17,18^ These two seeded growth methods have now been reported numerous times in the literature by Manners, Winnik, and other researchers, resulting in size controlled nanorods composed of a variety of materials ranging in length from 20 nm to >1 μm.^4,15,19–23^

Despite the high shape selectivity and precise control over NP size afforded by living CDSA, research into the biomedical applications of NPs prepared in this fashion is limited and largely focussed on the interaction of NPs with cancer cells *in vitro*.^19,24–29^ To produce crystalline-core micelles relevant for applications in nanomedicine the challenge lies with developing a system that results in NPs composed of biocompatible polymers that are colloidally stable in biological media. To ensure good colloidal stability a hydrophilic polymer must comprise the corona-forming block. With regard to the core-forming block, a crystallisable biocompatible polymer should be selected, with only a small number of promising examples based on polycaprolactone (PCL) and poly(l-lactide) (PLLA) currently present in the literature.^22,25–27^

Water dispersible crystalline core polymer micelles prepared to date are typically assembled in organic solvents and transferred into water by a solvent switch method such as dialysis.^30,31^ In a recent advancement of the field, Arno *et al.* reported the living CDSA of BCPs with a PCL core-forming block in an almost entirely aqueous environment.^25^ However, a small amount of organic solvent, eventually removed by evaporation, was required at both the spontaneous nucleation step and during seeded growth. Due to the hydrophobic nature of most crystallisable organic polymers, including PCL, there are currently no examples of ‘living’ CDSA in a purely aqueous environment. Such an assembly process is desirable as it would remove the need for the separation of organic solvents prior to biomedical application, and likely offer numerous advantages related to the cost and safety of particle synthesis scale-up. For this to be achieved, a BCP polymer with a hydrophilic corona-forming block and a crystallisable core-forming block with switchable solubility in water is likely to be required.

Hydrophilicity is not the only property of the corona-forming block that must be considered when pursuing applications of BCP micelles in the field of biomedicine. After intravenous injection NPs immediately encounter a wide range of circulating blood cells and plasma proteins which contribute to determining the fate of the particles. For example, phagocytic white blood cells, such as monocytes, granulocytes and dendritic cells, interact with foreign entities to eliminate them from systemic circulation. This is further supported by the adsorption of a plasma protein corona onto the NP, changing the surface chemistry and potentially causing an accelerated clearance by the immune system.^32–35^ To mitigate these effects low-fouling polymers are used as NP surface coatings.^36^ In this context poly(2-oxazoline)s (POx) have shown great promise.^36,37^

Functionality is incorporated into POx and their hydrophilicity tuned through variation of their amide bound sidechains.^38,39^ POx with methyl or ethyl substituents are highly water soluble, non-cytotoxic and exhibit protein repellent properties.^40–43^ Sidechain composition also influences POx crystallisation, with the materials with *n*-alkyl sidechains of length >C_4_ known to crystallise in the bulk.^44^ POx with propyl (C_3_) side chains show lower critical solution temperature (LCST) behaviour dependent on the conformation of this group, with poly(2-isopropyl-2-oxazoline) (P*i*PrOx) able to crystallise in water when heated above its cloud point temperature (*T*_CP_ of P*i*PrOx is 45–63 °C).^45–47^ POx therefore provide a unique opportunity for the one-pot preparation of biocompatible, thermoresponsive BCPs capable of undergoing CDSA simply through the combination of two different 2-oxazoline monomers. Despite this enormous potential, POx BCPs have only been discussed in detail as amphiphilic building blocks for spherical micelles and are largely unexplored in terms of their CDSA behaviour.^48^ Reports on the CDSA behaviour of POx to date have only described the preparation of hydrogels or ill-defined aggregates of fibre-like structures with poor colloidal stability, and a method for synthesising non-spherical POx particles of controlled size does not currently exist.^49–52^

In this report we combine the desirable biomaterial properties of POx with the exquisite control over NP size and shape afforded by living CDSA. This allowed for the first time the preparation of POx nanorods of tuneable length within a size range relevant for biomedical applications. The thermoresponsive nature of the crystallisable P*i*PrOx core-forming block used allowed the assembly of these structures to take place in an entirely aqueous environment. This represents the first example of the living CDSA method in the complete absence of organic solvents. Additionally, investigations into the biological interactions of this system were carried out in the form of both whole human blood immune cell association assays, and murine *ex vivo* biodistribution studies. Together, these experiments revealed that POx nanorods exhibit excellent stealth behaviour and sufficient blood circulation times to merit further in depth exploration of these materials as the basis for novel nanomedicines.

## Results and discussion

**Scheme 1:**
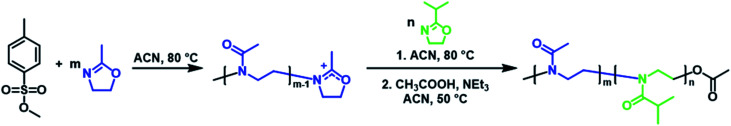
Synthesis of PMeOx-*b*-P*i*PrOx by sequential CROP of 2-methyl-2-oxazoline and 2-isopropyl-2-oxazoline initiated by methyl *p*-toluenesulfonate.

**Fig. 1:**
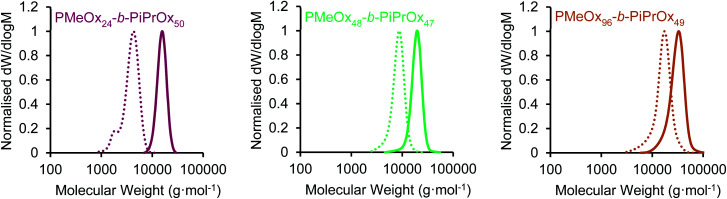
SEC chromatograms (RI, DMAc 0.03 wt% LiBr, 1 mL min^−1^) of PMeOx_*m*_-*b*-P*i*PrOx_*n*_ block copolymers (solid line) and PMeOx_*m*_ homopolymers (dashed line) characterised prior to *i*PrOx addition.

Three PMeOx-*b*-P*i*PrOx BCPs were prepared by the cationic ring-opening polymerisation (CROP) of 2-methyl-2-oxazoline (MeOx) and 2-isopropyl-2-oxazoline (*i*PrOx) in a sequential monomer addition approach (Scheme 1). PMeOx blocks with an average degree of polymerisation (DP_*n*_) of 25, 50 and 100 were targeted followed by a consistent P*i*PrOx DP_*n*_ of 50. Through the synthesis and study of BCPs with compositions ranging from a majority wt% of core-forming P*i*PrOx to a majority corona-forming PMeOx, we aim to provide a first insight into the effect of core : corona ratio on PMeOx-*b*-P*i*PrOx micelle morphology. All BCPs synthesised showed an excellent agreement with the targeted composition and narrow molar mass distributions with dispersities (*Đ*_M_) of <1.15 (Fig. 1 and S1†). To further confirm the composition of the BCPs, the materials were analysed by differential scanning calorimetry (DSC). In each case a small melt transition was observed at 185 °C and a larger melt at 195 °C (Fig. S2†).^53^ This observation is consistent with previous studies of the thermal properties of P*i*PrOx, thus indicating the presence of a crystallisable P*i*PrOx block in each of the copolymers.

To observe the cell association and *in vivo* biodistribution of PMeOx-*b*-P*i*PrOx micelles it was necessary to incorporate a fluorophore into the NP structure. For this role, cyanine5 (Cy5) was chosen due to its high quantum yield and emission in the red region of the visible spectrum where biological samples, especially blood, show low intensity autofluorescence. Our group has previously observed that Cy5 can influence the intracellular distribution of macromolecules,^54,55^ an effect we sought to minimise here by positioning the dye at the NP core–corona interface where it would be sterically shielded by the PMeOx corona. It has previously been demonstrated that functional polymer chain ends can be expressed on the surface of polymer crystals and similarly that dyes can be incorporated onto the surface of crystalline-core BCP NPs through the co-assembly of dye labelled and unlabelled crystallisable homopolymers.^56,57^ To achieve a similar result we synthesised a P*i*PrOx homopolymer end-functionalised with Cy5 (P*i*PrOx_40_-Cy5, Fig. S3†) as shown in Scheme S1.† The molecular weight characterisation of all polymers mentioned above is summarised in Table 1.

**Table 1:** Polymer molecular weights and composition data

Polymer	*M*_n,NMR_ a (g mol^−1^)	*M*_n,SEC_ b (g mol^−1^)	*Đ*_M_ b	wt% P*i*PrOx^c^
PMeOx_24_-*b*-P*i*PrOx_50_	6200	14 900	1.05	72
PMeOx_48_-*b*-P*i*PrOx_47_	9400	18 200	1.07	56
PMeOx_96_-*b*-P*i*PrOx_49_	13 400	28 200	1.13	40
P*i*PrOx_40_-Cy5	4900	5100	1.10	90

a Determined by ^1^H NMR (400 MHz, D_2_O). DP_*n*_ of PMeOx calculated based on monomer conversion and P*i*PrOx DP_*n*_ from the ratio of the integrals from the resonances of the methyl and isopropyl R-groups.

b SEC relative to PS standards (DMAc 0.03 wt% LiBr, 1 mL min^−1^).

c Calculated from block lengths determined by ^1^H NMR spectroscopic analysis.

BCP self-assembly was first investigated by dynamic light scattering to determine the *T*_CP_ of each BCP at 10 mg mL^−1^ in Milli-Q water (Fig. S4†). A *T*_CP_ of 53 °C was observed for PMeOx_24_-*b*-P*i*PrOx_50_ and a *T*_CP_ of 60 °C for both PMeOx_48_-*b*-P*i*PrOx_47_ and PMeOx_96_-*b*-P*i*PrOx_49_. All three BCPs appeared well-soluble in water below their *T*_CP_ and underwent a sharp transition from unimer to aggregates with a ∼20 nm number average hydrodynamic diameter upon heating. These structures are likely spherical micelles similar to those previously reported by Legros *et al.* when studying the self-assembly behaviour of PMeOx_50_-*b*-P*i*PrOx_50_.^50^

**Fig. 2:**
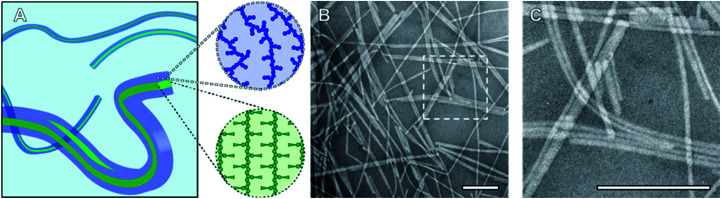
(A) Schematic diagram of fibre-like PMeOx_*m*_-*b*-P*i*PrOx_*n*_ nanorods showing crystalline P*i*PrOx core with a rectangular cross-section (green) and a solvated PMeOx corona (blue). (B) TEM image of PMeOx_48_-*b*-P*i*PrOx_47_ nanorods prepared by annealing a 2 mg mL^−1^ aqueous solution at 70 °C for 1 week. (C) Magnification of area outlined in (B). Scale bars equal to 200 nm.

To probe the CDSA behaviour of PMeOx_24_-*b*-P*i*PrOx_50_, PMeOx_48_-*b*-P*i*PrOx_47_ and PMeOx_96_-*b*-P*i*PrOx_49,_ solutions of each polymer in water were heated to 70 °C and NP formation analysed by transmission electron microscopy (TEM) after 24 h, 72 h and one week. To eliminate the need for sample dilution prior to TEM analysis concentrations of 1, 2 and 4 mg mL^−1^ were used for PMeOx_24_-*b*-P*i*PrOx_50_, PMeOx_48_-*b*-P*i*PrOx_47_ and PMeOx_96_-*b*-P*i*PrOx_49_ respectively. It was anticipated that at 70 °C, above the observed BCP *T*_CP_, the P*i*PrOx segments would become water-insoluble, aggregate and eventually crystallise forming the core of a NP, whilst the PMeOx blocks would remain solvated forming the NP corona as shown in Fig. 2A. In the case of PMeOx_24_-*b*-P*i*PrOx_50_ and PMeOx_48_-*b*-P*i*PrOx_47_, the polymer solutions became turbid within 24 h of annealing at 70 °C, and TEM analysis revealed the formation of fibre-like NPs (Fig. 2, S5 and S6B†). No change in particle morphology was observed after continued annealing for a total time of 1 week. The formed fibres had lengths ranging from ∼200 nm to <5 μm and core-widths between 20 to 50 nm. By TEM no aggregates were observed to form from PMeOx_96_-*b*-P*i*PrOx_49_ even after 72 h of annealing time. However, after annealing at 70 °C for 1 week the polymer solution became turbid and elongated NPs like those described above were observed (Fig. S5C and S6B†). Each of the three BCPs studied here formed high aspect ratio aggregates regardless of their composition. This is consistent with the anisotropic crystal growth observed for P*i*PrOx homopolymers and suggests that the formation of 2D lamella type structures driven by the crystallisation of this material will be challenging.^58^ Fibre-like crystalline-core micelles have previously been reported with circular, ellipsoidal and rectangular cross-sectional structures.^30,59,60^ Close examination of the TEM images of PMeOx_24_-*b*-P*i*PrOx_50_, PMeOx_48_-*b*-P*i*PrOx_47_ and PMeOx_96_-*b*-P*i*PrOx_49_ micelles revealed twists in the long axis of the fibres allowing the core to be visualised in multiple orientations (Fig. S7†). Throughout fibre rotation the edges of the NP core can clearly be visualised revealing an apparent change in core width upon twisting. This feature would not be present if the PMeOx_*m*_-*b*-P*i*PrOx_*n*_ micelles had a cylindrical shape, therefore the cross-sectional structure of these particles is likely rectangular (Fig. 2A).

Analysis of NP formation by TEM suggests that PMeOx_24_-*b*-P*i*PrOx_50_ and PMeOx_48_-*b*-P*i*PrOx_47_ form fibre-like NPs at a rate greater than PMeOx_96_-*b*-P*i*PrOx_49_. To investigate this hypothesis further, solutions of each BCP were prepared in D_2_O at the same concentrations as outlined above and annealed at 70 °C. During this process, P*i*PrOx solvation was monitored by ^1^H NMR spectroscopy.^61^ It was observed that the integration of the ^1^H resonance from the two CH_3_ groups (2.7–3.0 ppm) of the P*i*PrOx block of PMeOx_24_-*b*-P*i*PrOx_50_ decreased relative to the signal from the PMeOx methyl groups, at a greater rate than the other two BCPs. After just 24 h, peak integration had dropped to 9% of the value prior to annealing, with almost no further change after 72 h and 1 week (Table 2). Similar observations were made for PMeOx_48_-*b*-P*i*PrOx_47_, however the signal reduced at a slightly lesser rate and eventually by 87% after one week. In contrast, negligible change was observed for PMeOx_96_-*b*-P*i*PrOx_49_ after 72 h, and only a reduction of 60% was observed after 7 d of annealing at 70 °C. These results are consistent with TEM observations and show the rate of BCP self-assembly under the conditions measured can be ordered as follows, PMeOx_24_-*b*-P*i*PrOx_50_ > PMeOx_48_-*b*-P*i*PrOx_47_ ≫ PMeOx_96_-*b*-P*i*PrOx_49_. This trend reflects that the more hydrophilic the BCP, *i.e.* the higher the wt% PMeOx, the lower the rate of its CDSA. Finally, it should be noted that for each of the three BCPs the ^1^H NMR signals from the core-forming block do not completely disappear suggesting that some BCP remains molecularly dissolved even after 1 week of annealing at 70 °C.

**Table 2:** ^1^H NMR investigation into the rate of PMeOx_*m*_-*b*-P*i*PrOx_*n*_ self-assembly in D_2_O at 70 °C

Polymer	% Residual resonance from P*i*PrOx·2CH_3_ after annealing at 70 °C compared to BCP solution^a^		
	24 h	72 h	1 week
PMeOx_24_-*b*-P*i*PrOx_50_	9%	8%	6%
PMeOx_48_-*b*-P*i*PrOx_47_	21%	15%	13%
PMeOx_96_-*b*-P*i*PrOx_49_	98%	96%	40%

a P*i*PrOx·2CH_3_ resonance integral determined relative to PMeOx CH_3_ assuming no decrease in the NMR signal from the latter.

To probe the height of the core of the POx fibres, an aliquot from the colloidal solutions of the three BCPs was drop cast onto mica and analysed by AFM in the dried state (Fig. S8†). Interestingly, the fibres formed from the polymer containing the shortest corona forming block, PMeOx_24_-*b*-P*i*PrOx_50_, displayed the greatest height with a value of 11 nm (*n* = 20, standard deviation, *σ* = 1 nm), whereas fibres formed from PMeOx_48_-*b*-P*i*PrOx_47_ and PMeOx_96_-*b*-P*i*PrOx_49_ both displayed a height of 8 nm (*n* = 20, *σ* = 0.5 nm). As these values are significantly smaller than the *W*_*n*_ determined by TEM and the widths observed by AFM, we expect this value to reflect the smallest dimension (height) of the nanofibre core plus a small contribution from the collapsed PMeOx corona. Assuming that the P*i*PrOx backbone is parallel to the smallest dimension of the NP core, as is typical for polymer crystals,^62^ the core height will be determined by the P*i*PrOx chain length and average number of chain folds. An X-ray diffraction study of semi-crystalline POx by Litt *et al.* revealed the backbone length of one monomer unit in the P*i*PrOx crystal lattice to be 2.1 Å.^63^ Therefore, crystalline P*i*PrOx_50_ undergoing no chain-folds would have a backbone length of 10.5 nm. This value is in close agreement with the height of the PMeOx_24_-*b*-P*i*PrOx_50_ fibres, suggesting that little chain-folding takes place within their core. However, it is greater than the measured height of the PMeOx_48_-*b*-P*i*PrOx_47_ and PMeOx_96_-*b*-P*i*PrOx_49_ fibres indicating more P*i*PrOx chain-folding within the core of these particles. It is likely that fewer chain-folds are necessary to relieve the steric repulsion caused by dense grafting of PMeOx chains at the core–corona interface when the DP_*n*_ of this block is reduced from 50 or 100 to 25. Twists in the POx fibres were challenging to detect using AFM analysis, which could be due to a strong preference for PMeOx_*m*_-*b*-P*i*PrOx_*n*_ fibres to dry onto their largest face on the mica substrates used (Fig. S8†).

Final structural investigation of PMeOx_*m*_-*b*-P*i*PrOx_*n*_ fibres took the form of DSC and wide-angle X-ray scattering (WAXS) analysis of a freeze-dried aliquot of the PMeOx_48_-*b*-P*i*PrOx_47_ NP solution. The observed melt at 198 °C is in good agreement with our analysis of bulk PMeOx_48_-*b*-P*i*PrOx_47_ and the previously reported melting point of P*i*PrOx (Fig. S9A†).^53^ WAXS analysis revealed Bragg peaks with *d*-spacings of 10.8, 4.7 and 4.1 Å, characteristic of the 010, 100 and 110 lattice planes of P*i*PrOx (Fig. S9B†).^63^ This analysis confirms the presence of crystalline P*i*PrOx in the nanoparticle structure as shown in Fig. 2A, and that these BCP NPs fulfil the basic requirements for controlled CDSA.

To precisely tune the length of POx nanorods we developed a methodology for aqueous heat triggered seeded growth first focussing on NPs prepared from PMeOx_48_-*b*-P*i*PrOx_47_. The aqueous colloidal solution of PMeOx_48_-*b*-P*i*PrOx_47_ fibres prepared by spontaneous nucleation was cooled with an ice-bath and subjected to ultrasonication for 40 min. This resulted in a suspension of low size dispersity seed micelles with a number-average length (*L*_*n*_) and a standard deviation in length of 45 and 18 nm respectively, as determined by TEM analysis (Fig. S10†). A number-average width (*W*_*n*_) of 23 nm (*σ* = 5 nm) was also determined by TEM analysis (Fig. S10C†). In an attempt to prepare POx nanorods with a *L*_*n*_ of 90 nm, one mass equivalent of PMeOx_48_-*b*-P*i*PrOx_47_ unimer was added as a 1 mg mL^−1^ aqueous solution to an aliquot of seeds at the same concentration. This mixture was then heated to 70 °C for 16 h. This led to slight visible polymer agglomeration and sedimentation, therefore the experiment was repeated reducing the annealing temperature to 60 °C (Fig. S11†). No precipitation was observed and the resulting NPs were analysed by TEM. It is at this point that our current study differs from other literature reports of seeded CDSA. Surprisingly, the *L*_*n*_ of the observed elongated nanorods was 263 nm, close to six times the length of the seed micelles used (Fig. S12†). This unexpected magnitude of particle elongation could potentially be caused by end-to-end rod fusing or a decrease in linear aggregation number during the seeded growth process. However, as NP length dispersity (as defined by weight average length/*L*_*n*_, *L*_*w*_/*L*_*n*_) remained low (1.08), the histogram of measured rod lengths showed a narrow monomodal distribution, and there was no detectable change in rod width, these two outcomes can be excluded (Fig. S12B†).

**Fig. 3:**
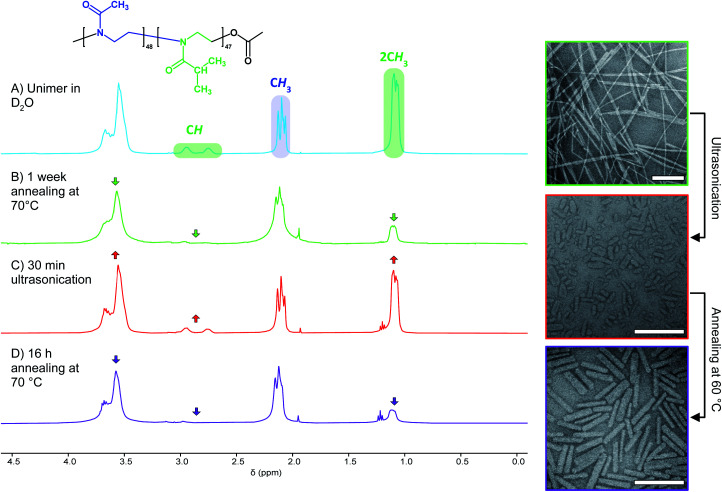
Concomitant nanofibre fragmentation and BCP dissolution. Left, ^1^H NMR spectra (400 MHz, 2 mg mL^−1^ in D_2_O) of PMeOx_48_-*b*-P*i*PrOx_47_ unimer (A), nanofibres prepared annealing the BCP solution at 70 °C for 1 week (B), a mixture of seed fragments and BCP unimer prepared *via* ultrasonication (C) and elongated seeds (D). Right, TEM images showing polydisperse nanofibres (top), seeds (middle) and elongated seeds (bottom). Scale bars equal to 250 nm.

In search of an alternative explanation we annealed a solution of seed micelles at 60 °C without any addition of further BCP and observed that nanorod *L*_*n*_ rose from 45 to 123 nm. The *W*_*n*_ of the annealed and elongated particles was observed by TEM to be 27 nm (*σ* = 6 nm), an increase of 4 nm compared to the NPs before annealing (Fig. S12C†). This is an increase of less than one standard deviation and is likely within the standard error of the measurement. The threefold length increase implies that the NP solution after sonication contains a mixture of seed micelles and unimer and, more specifically, that the molar ratio of BCP in solution to that comprising a nanorod is 2 : 1. Without the addition of further BCP unimer the polymer present in solution must be the result of NP disassembly. To confirm this, we examined the ^1^H NMR spectra of PMeOx_48_-*b*-P*i*PrOx_47_ micelles in D_2_O before and after ultrasound treatment. This revealed an increase in the integral of the resonance from the P*i*PrOx·2CH_3_ protons relative to that from PMeOx·CH_3_ protons, consistent with concomitant NP fragmentation and dissolution (Fig. 3B and C). In accordance with the nanorod growth as observed by TEM, the P*i*PrOx ^1^H signals then decrease again upon annealing of the unimer–seed mixture at 60 °C (Fig. 3D). These observations closely resemble the self-seeding process, however, due to the thermoresponsive nature of P*i*PrOx, unimer is generated by dissolving seeds at low temperature and growth triggered by heating.

**Fig. 4:**
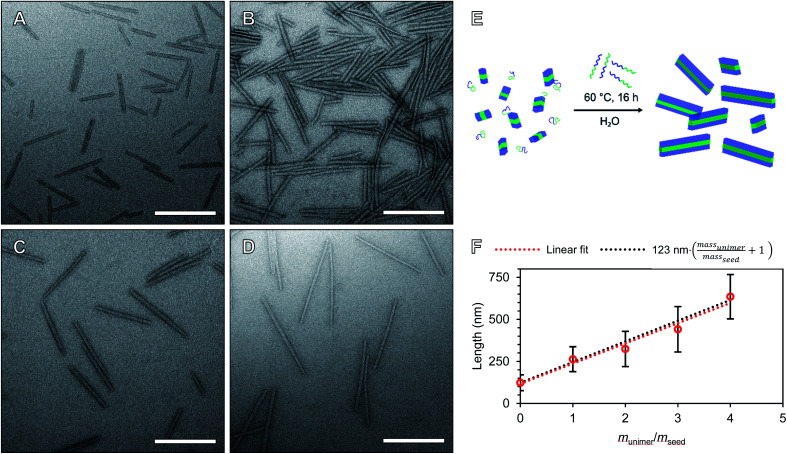
Preparation of length controlled POx nanorods by seeded growth in water. TEM images of length controlled POx nanorods prepared by seeded growth from 2 (A), 3 (B), 4 (C) and 5 (D) mass equivalents of PMeOx_48_-*b*-P*i*PrOx_47_. (E) Schematic diagram of the seeded growth procedure in fully aqueous environment. (F) Plot of unimer to seed ratio (*m*_unimer_/*m*_seed_) against particle *L*_*n*_. Error bars indicate standard deviation in rod length. Samples were stained with UranyLess prior to imaging. Scale bars equal to 250 nm.

We will define the length of PMeOx_48_-*b*-P*i*PrOx_47_ seeds as the value after annealing at 60 °C, 123 nm, as consistent with a living CDSA process, this value is close to half the *L*_*n*_ observed when PMeOx_48_-*b*-P*i*PrOx_47_ nanorods were prepared *via* the addition of one mass equivalent of BCP unimer (263 nm). Length controlled nanorods were then prepared *via* seeded growth by combining aqueous solutions of BCP unimer and seed micelle–unimer mixture. To build upon the seeded growth outlined earlier, 2, 3 and 4 mass equivalents of PMeOx_48_-*b*-P*i*PrOx_47_ were added as a 1 mg mL^−1^ aqueous unimer solution to portions of the seed solution at the same concentration. Analysis of these mixtures revealed no detectable NP elongation after storing the solutions at 23 °C for 72 h due to the hydrophilicity of the added BCP at this temperature. However, when the samples were heated to 60 °C for 16 h epitaxial growth of the added polymer was triggered. TEM analysis revealed elongated nanorods with *L*_*n*_ values ranging from 324 to 635 nm and length dispersities ≤1.10 (Fig. 4). The particle length analysis is summarised in Table S2† and length histograms shown in Fig. S13.† Plotting *L*_*n*_ against mass equivalents of PMeOx_48_-*b*-P*i*PrOx_47_ reveals a linear relationship between these parameters and a gradient of 120 nm per equivalent (Fig. 4F). This value is in close agreement with the length of the annealed seed micelles consistent with living seeded growth. To confirm the presence of crystalline P*i*PrOx in the grown nanorods, a freeze-dried aliquot of a sample prepared with a *m*_unimer_/*m*_seed_ of 4 was subjected to WAXS analysis. Bragg peaks matching those reported in the literature and those observed in the parent polydisperse fibres were detected, supporting our proposed living CDSA NP growth mechanism (Fig. S14†). These results represent the first example of the CDSA process occurring in an entirely aqueous environment and in response to increased temperature.

The preparation of POx nanorods of controlled length from PMeOx_24_-*b*-P*i*PrOx_50_ was also attempted. Seed micelles were prepared by ultrasonication of first generation PMeOx_24_-*b*-P*i*PrOx_50_ fibres resulting in a suspension of short nanorods with a *L*_*n*_ of 56 nm (*L*_*w*_/*L*_*n*_ = 1.17, Fig. S15A†). 0, 1, 2, 3 or 4 mass equivalents of PMeOx_24_-*b*-P*i*PrOx_50_ unimer were added to diluted suspensions of these seeds to give a final concentration of 0.5 or 0.25 mg mL^−1^. These mixtures were then heated to 60 °C for 16 h which in each case resulted in a slightly turbid suspension. TEM analysis of the samples revealed nanorod growth had occurred but also that the particle's cores appeared to fuse together as shown in Fig. S15B and C.† This suggests that the shorter PMeOx_25_ corona does not provide sufficient steric shielding of the P*i*PrOx core to prevent nanorod fusing during annealing. As such, the preparation of PMeOx_24_-*b*-P*i*PrOx_50_ nanorods was not further pursued. Similar fusion of crystalline-core polymer nanoparticles has previously been observed between cylindrical micelles that include sections with no corona-forming block and those that have had their corona cleaved post-assembly.^64,65^ At this stage we did not attempt to synthesise PMeOx_96_-*b*-P*i*PrOx_49_ NPs by seeded growth due to the low rate of self-assembly observed for this polymer during spontaneous nucleation.

Having established that NP disassembly occurs during seed micelle preparation we sought to investigate the kinetic stability of crystalline-core PMeOx_48_-*b*-P*i*PrOx_47_ micelles generated by seeded growth. For this purpose, a 1 mg mL^−1^ D_2_O solution of nanorods with a *L*_*n*_ of 275 nm was prepared. TEM analysis immediately after seeded growth revealed a narrow monomodal length distribution and a *L*_*w*_/*L*_*n*_ of 1.08 (Fig. S16†). The nanorod solution was split into two aliquots, one was stored at 23 °C and the other at 37 °C, and the effect of aging monitored by ^1^H NMR spectroscopy and TEM (Fig. S17†). After 6 h, there was no detectable increase in the intensity of the ^1^H resonance from the *i*PrOx·2CH_3_ groups in either sample, and no change was observed in the nanorod *L*_*n*_ or *L*_*w*_/*L*_*n*_. This shows that the nanorods are kinetically stable over this period. After 24 h at 23 °C a minor increase in the ^1^H resonance from the P*i*PrOx sidechain compared to that of the PMeOx block was observed (Fig. S17A†), and *L*_*n*_ reduced slightly to 259 nm (*L*_*w*_/*L*_*n*_ = 1.11). No decrease in *L*_*n*_ or increase of *L*_*w*_/*L*_*n*_ was observed for the sample stored at 37 °C. Continued monitoring of both samples over a 5 d period revealed, a gradual increase of the ^1^H resonances associated with the P*i*PrOx block over time, suggesting that slow NP dissolution eventually occurred under these conditions (Fig. S17A†). TEM analysis after 5 d revealed that the *L*_*n*_ of the sample stored at 23 °C decreased to 237 nm (Fig. S17B†). Furthermore, *L*_*w*_/*L*_*n*_ increased to 1.17 and a low length shoulder was visible in the length distribution histogram. Significantly less particle dissolution was observed by NMR spectroscopy for the sample stored at 37 °C. Consistent with this observation, nanorod *L*_*n*_ decreased by 15 nm only, and *L*_*w*_/*L*_*n*_ increased to 1.10 (Fig. S17C†). These results show that when stored below the LCST of the core-forming block, PMeOx_48_-*b*-P*i*PrOx_47_ nanorods are not kinetically trapped structures as is typical for NPs formed by CDSA.

Post intravenous administration, opsonization of nanomaterials by circulating plasma proteins can lead to increased phagocytosis and premature clearance from the body. This necessitates engineering ‘stealth’ materials to limit fouling and avoid detection by phagocytic immune cells *e.g.* neutrophils, monocytes, and dendritic cells.^66^ To allow for *in vitro* cell association to be determined, a Cy5 dye was incorporated into the POx nanorod structure. This was achieved by co-assembling P*i*PrOx_40_-Cy5 and PMeOx_48_-*b*-P*i*PrOx_47_ unimers through simple mixing of unimeric solutions of the two BCPs with seeds prior initiating NP growth at elevated temperature. Incorporation of Cy5 *via* the *ω*-terminus of a P*i*PrOx_40_ homopolymer ensured its positioning either within the nanorod core or more likely, at the nanorod core : corona interface. At both possible locations we anticipate that steric shielding by the PMeOx corona would minimise dye influence on the bio-nano interactions of the particles. The zeta potential of POx nanorods prepared by seeded growth both with and without incorporation of P*i*PrOx_40_-Cy5 was measured in Milli-Q water returning values of −4.5 and −3.0 mV respectively (Fig. S18†). These near zero zeta potentials are comparable to those of PEGylated nanoparticles and are expected to minimise the adsorption of serum proteins that can increase the likelihood of phagocytosis.^67^

**Fig. 5:**
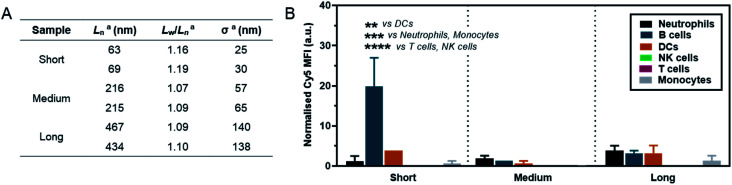
(A) Summary of length analysis for Cy5-labelled POx nanorod used in immune cell association assay. ^*a*^Determined by TEM analysis. (B) Normalised Cy5 mean fluorescence intensity (MFI) associated with different immune cell types in whole blood after incubation with POx-Cy5 nanorods for 1 h. Error bars represent standard error of mean (*n* = 2); ****p < 0.0001, ***p < 0.001, **p < 0.01, two-way ANOVA with Sidak's correction for multiple comparisons.

As a preliminary investigation into the stealth behaviour of PMeOx-*b*-P*i*PrOx nanorods, a human blood immune cell association assay was carried out.^66^ This assay was repeated twice on different days with two batches of Cy5-labelled ‘short’ (*L*_*n*_ = 63 and 69 nm, *W*_*n*_ = 26 and 26 nm), ‘medium’ (*L*_*n*_ = 216 and 215 nm, *W*_*n*_ = 27 and 26 nm) and ‘long’ (*L*_*n*_ = 467 and 434 nm, *W*_*n*_ = 29 and 28 nm) nanorods, which were prepared separately immediately prior to cell incubation. The incorporation of P*i*PrOx_40_-Cy5 into the NPs was not observed to affect their width and between batches variation in nanorod *L*_*n*_ and *L*_*w*_/*L*_*n*_ was low. Length analysis summarised in Fig. 5A and length distribution histograms shown in Fig. S19,† demonstrate the excellent reproducibility of the NP synthesis. POx nanorods were incubated separately with both whole blood and plasma-stripped ‘washed’ blood at 10 μg mL^−1^ for one hour at 37 °C. Nanorod cell association was then analysed by flow cytometry with data expressed as the median fluorescence intensity (MFI) from Cy5 associated with a given immune cell population (Fig. 5 and S20†), and as the percentage of immune cells within each population with a detectable level of Cy5 fluorescence (Fig. S21†). The flow cytometry gating strategy is summarised in Fig. S22.†

The short nanorods demonstrated a modest yet statistically significant localisation to B cells, displayed both in terms of percentage of B cell population identified as nanorod-positive (7%, Fig. S21A†), and normalised B cell Cy5 MFI (20, Fig. 5). Increasing nanorod length appeared to result in a reduction in B cell localisation, with both the medium and long samples recording <1% nanorod-positive B cell populations and a Cy5 MFI of <5. The low-level association of the short nanorods with B cells was not observed in the absence of plasma (Fig. S20 and S21B†). This could correlate with low levels of complement protein adsorption facilitating nanorod-B cell association as previously shown for other NP technologies.^68^ In general, all nanorods displayed low levels of association across all phagocytic cell types (neutrophils, monocytes, DCs), correlating with a low Cy5 MFI and indicative of transient NP–cell interactions. This stealth behaviour is likely afforded by the presence of the PMeOx corona of the nanorods, and suggests that these NPs are a promising platform upon which to base the design of nanomedicines that require administration *via* the blood stream. Proteomic analysis of the biomolecular corona formed on POx nanorods in human serum will form the basis of a future study of these materials. It is hoped that the resulting information would allow for a direct comparison between the stealth behaviour of POx nanorods and that of benchmark PEGylated NPs.

Stealth properties were shown to be afforded by not only the chemical composition of the nanorods but also their structure. Exposure of unimeric PMeOx_48_-*b*-P*i*PrOx_39_-Cy5 (synthesised by reaction of PMeOx_48_-*b*-P*i*PrOx_39_-NH_2_ with Cy5-NHS, Scheme S2† and Fig. S23†) to whole and washed blood resulted in significant association with every cell type examined, with particularly high localisation to phagocytic monocytes and neutrophils (Fig. S24†).

**Fig. 6:**
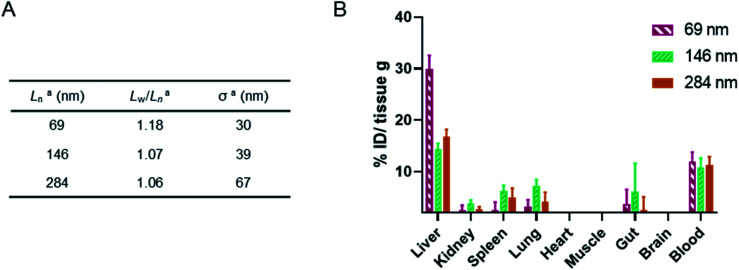
(A) Summary of length analysis for Cy5-labelled POx nanorod used in murine *ex vivo* biodistribution study. ^*a*^Determined by TEM analysis. (B) Murine biodistribution of NR1, NR2 and NR3. Concentration in significant organs measured by fluorescence signal *ex vivo* 24 h post single injection (*n* = 3, mean ± SEM).

Finally, a murine *ex vivo* biodistribution study was performed to assess systemic circulation lifetime and the clearance mechanism of the PMeOx_48_-*b*-P*i*PrOx_47_ nanorods. Again three Cy5-labelled nanorod samples of differing *L*_*n*_ were prepared, however, samples were split over a narrower length range due to concerns about the passage of long nanorods through the mouse circulatory system. Samples with *L*_*n*_ of 69, 146 and 284 nm were studied, with length analysis summarised in Fig. 6A and length distribution histograms shown in Fig. S25.† To allow injection of sufficient material into the animal models using a final injection volume of 100 μL, the concentration of the labelled nanorods was increased to 10 mg mL^−1^ by centrifugal filtration using a concentrator with a 30 kDa molecular weight cut-off membrane. During this process it was observed that the filtrate in each case was colourless indicating that no free Cy5 or P*i*PrOx_40_-Cy5 unimer is present in the nanorod colloidal solutions prior to injection. This is crucial as free Cy5 dye or unimeric P*i*PrOx_40_-Cy5 would be indistinguishable from Cy5-labelled POx nanorods during *in vitro* or *ex vivo* analysis.

24 h post injection the heart, brain, stomach, liver, spleen, kidney and lung were collected from each animal along with the blood and a muscle sample (Fig. 6, and S26†). The fluorescence signal from each was determined and converted to percentage of injected dose per gram of tissue (% ID g^−1^). As shown in Fig. 6, the three nanorod samples displayed a similarly low accumulation in kidney, spleen and lung tissue. In each case the % ID g^−1^ for these organs was <10%. Significantly higher liver uptake 24 h post injection, nearly 30% ID g^−1^, was observed for the shortest nanorods compared to that from the longer samples, which displayed a liver uptake of ∼15% ID g^−1^. These results suggest that the major clearance pathway for the POx nanorods is through the liver. The fluorescence from the blood pool is comparable for all three samples, with over 10% ID remaining in the blood stream 24 h post injection (Fig. 6). This value is encouraging and again suggests that the composition, shape and size of the POx nanorods presented here is compatible with delivery by intravenous administration.

## Conclusion

In summary, we produced three PMeOx_*m*_-*b*-P*i*PrOx_*n*_ BCPs, where *m* = 24, 48 or 96 and *n* = ∼50. We showed each polymer undergoes thermally induced CDSA to form fibres, with a rate of self-assembly proportional to the overall hydrophobicity of the polymer. The prevalence of the same high aspect ratio morphology across all three samples regardless of core : corona ratio indicates a strong preference of P*i*PrOx for 1D crystallisation. We were able to tune the *L*_*n*_ of PMeOx_48_-*b*-P*i*PrOx_47_ nanorods up to a *L*_*n*_ of 635 nm using the living CDSA methodology. This self-assembly process was carried out in an entirely aqueous environment for the first time. Interestingly, we observed simultaneous NP fragmentation and dissolution upon fibre sonication, and slow temperature dependant disassembly of POx nanorods stored in water. These observations represent key differences between heat-triggered CDSA and conventional CDSA methods. After preparing a series of nanorods which incorporate Cy5 at the core–corona interface, we were able to investigate these novel materials in a biological context. We observed low association with white blood cells, consistent with the known stealth behaviour of POx based materials, promising blood circulation lifetimes in mice and systemic clearance mainly *via* the liver. Little effect of POx nanorod length within the range of 63–467 nm under the conditions investigated was detected. These findings form a promising base from which to build improved nanomedicines that exploit the low fouling and versatile chemistry of POx, and the control over NP size and shape afforded by CDSA.

## Experimental

Experimental details are listed in the accompanying ESI† document.

## Author contributions

J. R. F. and K. K. conceived and designed the experiments. J. R. F. carried out and analysed polymer synthesis and self-assembly, E. H. P. the human blood immune cell association assays, K. A. the *ex vivo* biodistribution study and M. A. R. the AFM analysis. The manuscript was prepared by J. R. F. and K. K. and edited by all authors. Acquisition of the financial support for the project leading to this publication was secured by K. K., T. P. D. and S. K.

## Conflicts of interest

The authors report no conflicts of interest.

## Supplementary Material

SC-012-D1SC00938A-s001
