# Supplementary material for: Stealth nanorods via the aqueous living crystallisation-driven self-assembly of poly(2-oxazoline)s
Source: Chem Sci. 2021 Apr 12;12(21):7350–60. doi: 10.1039/d1sc00938a (PMC8171341; doi:10.1039/d1sc00938a)
Supplement: SC-012-D1SC00938A-s001 [file SC-012-D1SC00938A-s001.pdf]

Electronic Supplementary Material (ESI) for Chemical Science.
This journal is © The Royal Society of Chemistry 2021

SUPPORTING INFORMATION

Stealth Nanorods via the Aqueous Living Crystallisation-Driven Self-Assembly of Poly(2-oxazoline)s

John R. Finnegan,[a] Emily H. Pilkington,[a],[b] Karen Alt,[c] Md. Arifur Rahim,[d] Stephen J. Kent,[b] Thomas P. Davis*[a],[e] and Kristian Kempe*[a],[f]

[a] ARC Centre of Excellence in Convergent Bio-Nano Science and Technology, Drug Delivery, Disposition and Dynamics, Monash Institute of Pharmaceutical Sciences, Monash University, Parkville, Victoria 3052, Australia

[b] ARC Centre of Excellence in Convergent Bio-Nano Science, Department of Microbiology and Immunology, Peter Doherty Institute for Infection and Immunity, The University of Melbourne, Parkville, Victoria 3010, Australia

[c] NanoTheranostics Laboratory, Australian Centre for Blood Diseases, Monash University, Melbourne, Victoria 3004, Australia

[d] School of Chemical Engineering, University of New South Wales (UNSW), Sydney, NSW 2052, Australia

[e] Australian Institute for Bioengineering and Nanotechnology, The University of Queensland, Brisbane, QLD 4072, Australia

[f] Materials Science and Engineering, Monash University, Clayton, VIC 3800, Australia

Contents

Materials and Methods 2
Synthetic Procedures 3
Self-Assembly Procedures 5
Biology 6
Supporting Schemes and Tables 7
Supporting Figures 10
References 23

Materials and Methods

All moisture-sensitive reactions were carried out in oven (100 °C) dried glassware under a positive nitrogen pressure using standard Schlenk techniques. All compounds were purchased from Sigma-Aldrich and used without further purification unless stated otherwise. Methyl *p*-toluenesulfonate was purified by fractional distillation under reduced pressure. Anhydrous acetonitrile was stored over activated 3 Å molecular sieves prior to use. 2-Methyl-2-oxazoline was purified by distillation over BaO. 2-Isopropyl-2-oxazoline (*i*PrOx) was synthesised following the procedure outlined in ref. [1] and purified by distillation over BaO. Cyanine5-NHS ester was purchased from Lumiprobe.

Nuclear magnetic resonance (NMR) spectra were recorded on a Bruker Avance III 400 MHz spectrometer using an external lock and referenced internally to a resonance from residual protonated solvent. Chemical shifts (δ) are reported in parts per million (ppm). NMR solvents ($CDCl_3$ and D_2O) were purchased from Cambridge Isotope Laboratories, Inc and used as received.

Size-exclusion chromatography (SEC) analyses of polymer samples were performed using a Shimadzu modular system comprising a DGU-20A3R degasser unit, an SIL-20A HT autosampler, a 10.0 µm bead-size guard column (50 × 7.8 mm) followed by three KF-805L columns (300 × 8 mm, bead size: 10 µm, pore size maximum: 5000 Å), a SPD-20A UV/Vis detector, and an RID-10A differential refractive-index detector. The temperature of columns was maintained at 40 °C using a CTO- 20A oven. The eluent was 0.03 wt% LiBr in *N*,*N*-dimethylacetamide (DMAc, CHROMASOLV Plus for HPLC) and the flow rate kept at 1.0 mL/min using a LC-20AD pump. A molecular weight calibration curve was produced using commercial narrow molecular weight distribution polystyrene standards with molecular weights ranging from 500 to 2 × 10^6 Da. Polymer solutions were analysed at 2 mg/mL after filtration through 0.45 µm PTFE membrane.

Dynamic light scattering (DLS) and zetapotential measurements were performed using a Malvern Zetasizer Nano ZS. Cloud point temperature was determined by DLS analysis of filtered 10 mg/mL polymer solutions in Milli-Q water in a disposable sizing cuvette (Malvern, Zen0040). Polymer solutions were heated in 2 °C increments from 30 to 70 °C, equilibrating for 120 s and recording 3 measure of at least 10 runs at each temperature. Zetapotential measurements were carried out at 25 °C on unfiltered 1 mg/mL nanorod suspensions in Milli-Q water in a disposable folded capillary cuvette (Malvern, DTS1070). Values shown represent the mean and standard deviation of three measurements comprised of a minimum of ten runs each.

Transmission electron microscopy (TEM) was performed using either a Tecnai F20 or a Thermo Scientific Talos L120C microscope, both instruments were operated at 200 kV. Samples for TEM analysis were prepared by cooling polymer suspensions to room temperature followed by the addition of 5 µL of colloidal solution to a negative glow discharge treated carbon coated copper grid (ProSciTech). After approximately 60 s excess sample solution was removed by contact with filter paper. Samples to be stained with UranyLess (Electron Microscopy Sciences) were then placed onto a single drop of UranyLess solution for 50 s. Excess stain solution was removed by contact with filter paper and the grid allowed to air dry prior to imaging.

Analysis of TEM images was carried out using ImageJ software (National Institute of Health). Number average (L_n) and weight average particle length (L_w) were calculated using the equations below.

$$L_n = \frac{\sum n_i L_i}{\sum n_i}, L_w = \frac{\sum n_i L_i^2}{\sum n_i L_i}, W_n = \frac{\sum n_i W_i}{\sum n_i}$$

Atomic force microscopy (AFM) was performed using a Bruker dimension icon SPM (USA). A triangular silicon nitride cantilever (ScanAsyst Air probe, spring constant 0.4 N/m, Bruker) with a tip radius of 2–10 nm was used for the topographic analysis. Samples were scanned with the PeakForce Tapping mode with a scan rate of 0.70 Hz. While scanning, the peak force and feedback gain were dynamically optimized by the ScanAsyst auto control. NanoScope Analysis 2.0 software was used to process the raw data and height analyses. Samples were prepared by adding 50 µL of nanoparticle solution to freshly cleaved mica. Excess solvent was removed by blotting with filter paper and the substrate dried under N_2 flow.

Differential scanning calorimetry (DSC) was performed using a PerkinElmer DSC8500 coupled to a PerkinElmer Intracooler 2 cooling system at a scanning rate of 10 °C·min^{-1}. Samples were sealed in 50 µL aluminium pans featuring a pinhole lid.

X-ray diffraction was carried out using a Bruker D8 Advance with Cu K-alpha radiation (λ = 1.5406 Å) and Lynxeye XE position sensitive detector running in 1D mode. Scatter was collected using Bragg-Brentano reflection geometry, a viable divergence slit was used with a constant sample illumination width at 17 mm. Diffraction peak positions and d-spacings were determined using Bruker DIFFRAC.EVA software.

Sonication. A Sonics VC 505 ultrasonic processor was used fitted with a 1/8 " tapered probe.

Synthetic Procedures

Poly(2-methyl-2-oxazoline)$_n$-*block*-poly(2-isopropyl-2-oxazoline)$_m$ (PMeOx$_n$-*b*-P*i*PrOx$_m$, where *n* and *m* represent average degree of polymerisation) were synthesised following the general procedure outlined below for PMeOx$_{48}$-*b*-P*i*PrOx$_{47}$.

PMeOx$_{48}$-*b*-P*i*PrOx$_{47}$. Methyl *p*-toluenesulfonate (35.6 µL, 0.24 mmol, 1 eq), anhydrous acetonitrile (4.9 mL) and 2-methyl-2-oxazoline (1.0 mL, 12 mmol, 50 eq) were transferred under nitrogen to an oven-dried greaseless Schlenk flask equipped with magnetic stirrer. The flask was then sealed and the reaction mixture heated with stirring to 80 °C for 7.5 h. A 0.3 mL aliquot was removed from the reaction mixture for molecular weight analysis of the PMeOx block, then 2-isopropyl-2-oxazoline (1.3 mL, 11 mmol, 50 eq) added, the flask sealed and the reaction mixture stirred at 80 °C for a further 16 h. To quench the polymerisation acetic acid (64 µL, 1.1 mmol, 5 eq) and triethylamine (156 µL, 1.1 mmol, 5 eq) were added and the reaction mixture stirred for 5 h at 60 °C. The resulting block copolymer was then precipitated directly into diethyl ether, followed by two subsequent precipitations from CH_2Cl_2 into diethyl ether. The resulting colourless solid was dialysed against water for 3 d using 12-14 kDa MWCO dialysis tubing (Cellu-Sep T3) and isolated by freeze-drying. Yield = 1.3 g (59%)

^{1}H NMR (400 MHz, D_2O) δ: 4.33 – 4.25 (s, CONC*H*$_2$, 2H), 4.21 – 3.08 (m, NC*H*$_2$C*H*$_2$, 378H), 3.10 – 2.60 (d, CH_3C*H*CH_3, 47H), 2.26 – 1.91 (m, COC*H*$_3$, 144H), 1.56 – 0.67 (m, 2C*H*$_3$, 282H).
SEC (RI, DMAc): M_n ($Đ_m$) = 18,200 Da (1.07)

PMeOx$_{24}$-*b*-P*i*PrOx$_{50}$. Yield = 1.2 g (71%)
^{1}H NMR (400 MHz, D_2O) δ: 4.33 – 4.25 (s, CONC*H*$_2$, 2H), 4.20 – 3.14 (m, NC*H*$_2$C*H*$_2$, 294H), 3.09 – 2.60 (d, CH_3C*H*CH_3, 50H), 2.46 – 1.87 (m, COC*H*$_3$, 72H), 1.48 – 0.73 (m, 2C*H*$_3$, 300H).
SEC (RI, DMAc): M_n ($Đ_m$) = 14,900 Da (1.05)

PMeOx$_{96}$-*b*-P*i*PrOx$_{49}$. Yield = 2.5 g (65%)
^{1}H NMR (400 MHz, D_2O) δ: 4.33 – 4.25 (s, CONC*H*$_2$, 2H), 4.10 – 3.14 (m, NC*H*$_2$C*H*$_2$, 578H), 3.04 – 2.63 (d, CH_3C*H*CH_3, 49H), 2.45 – 1.80 (m, COC*H*$_3$, 288H), 1.50 – 0.70 (m, 2C*H*$_3$, 294H).
SEC (RI, DMAc): M_n ($Đ_m$) = 28,200 Da (1.13)

P*i*PrOx$_{40}$-phthalimide. Methyl *p*-toluenesulfonate (50.7 µL, 0.3 mmol, 1 eq), anhydrous acetonitrile (6.3 mL) and 2-isopropyl-2-oxazoline (2.0 mL, 16.8 mmol, 50 eq) were transferred under nitrogen to an oven-dried greaseless Schlenk flask equipped with magnetic stirrer. The flask was then sealed and the reaction mixture heated to 80 °C for 10.5 h. To quench the reaction potassium phthalimide (0.080 g, 0.43 mmol, 1.4 eq) was added and the reaction mixture stirred at 70 °C for 14 h. Solids were removed by filtration and volatiles removed under vacuum. The resulting residue was dissolved in CH_2Cl_2, washed twice with saturated $NaHCO_3$, and once with brine then dried over $MgSO_4$. The volume of the solution was reduced under vacuum and P*i*PrOx$_{39}$-phthalimide precipitated into petroleum benzine. After a further precipitation P*i*PrOx$_{39}$-phthalimide was isolated as a white solid. Yield = 1.5 g (79%)
^{1}H NMR (400 MHz, $CDCl_3$) δ: 8.00 – 7.60 (m, Ar, 4H), 3.96 – 3.79 (m, C*H*$_2$N2CO, 2H), 3.79 – 3.15 (m, NC*H*$_2$C*H*$_2$, 158H), 3.04 – 2.50 (d, CH_3C*H*CH_3, 40H), 1.48 – 0.73 (m, 2C*H*$_3$, 240H).

P*i*PrOx$_{40}$-NH$_2$. In a 50 mL round-bottom flask fitted with magnetic stirrer P*i*PrOx$_{40}$-phthalimide (1.0 g, 0.21 mmol, 1 eq) was dissolved in ethanol (25 mL) followed by the addition of hydrazine monohydrate (162 µL, 3.3 mmol, 16 eq). The reaction mixture was then heated to reflux for 22 h, cooled to room temperature and filtered. Subsequently, 5 M HCl (aq) was added to adjust the pH value of the solution to 2 and the resulting precipitate removed by filtration. Volatiles were then removed under vacuum and the residue dissolved in water. Saturated NaOH (aq) was added to increase the pH value of the solution to 10. This solution was then extracted with CH_2Cl_2 and washed three times with water. The organic layer was dried over $MgSO_4$ and concentrated under vacuum. P*i*PrOx$_{40}$-NH$_2$ was isolated by precipitation into petroleum benzine. Yield = 410 mg (41%)
^{1}H NMR (400 MHz, $CDCl_3$) δ: 3.79 – 3.15 (m, NC*H*$_2$C*H*$_2$, 160H), 3.04 – 2.50 (d, CH_3C*H*CH_3, 40H), 1.48 – 0.73 (m, 2C*H*$_3$, 240H).
SEC (RI, DMAc): M_n ($Đ_m$) = 5,060 Da (1.10)

P*i*PrOx$_{40}$-Cy5. In an oven-dried greaseless Schlenk flask, P*i*PrOx$_{40}$-NH$_2$ (25 mg, 0.006 mmol, 1 eq), Cy5-NHS (8 mg, 0.013 mmol, 2.2 eq) and triethylamine (100 µL, 0.717 mmol, 120 eq) were dissolved in anhydrous *N*,*N*-dimethylformamide (1.5 mL). The reaction mixture was stirred under nitrogen in absence of light at room temperature for 14 h. Volatiles were removed under vacuum and the residue was re-dissolved in 5.0 mL of $CHCl_3$. Excess Cy5 was removed by preparative size exclusion column chromatography (Bio-BeadsTM S-X1, 600 - 14000 MW exclusion range) and P*i*PrOx$_{40}$-Cy5 isolated as a blue solid by freeze-drying. Yield = 24 mg (96%).
^{1}H NMR (400 MHz, D_2O) δ: 8.16 – 7.93 (br, C*H*CHC*H*, 2H), 7.68 – 7.20 (m, ArH, 8H), 6.71 – 6.48 (br, CHC*H*CH, H), 6.36 – 6.16 (br, 2CC*H*CH, 2H), 4.56 – 4.37 (br, NHC*H*$_2$CH$_2$N, 2H), 3.96 – 3.16 (m, NC*H*$_2$C*H*$_2$, 158H), 2.86 (d, CH_3C*H*CH_3, 40H), 1.74 – 1.58 (s, 4C*H*$_3$, 12H), 1.42 – 0.74 (m, 2C*H*$_3$, 240H).
SEC (RI, DMAc): M_n ($Đ_m$) = 4,444 Da (1.12)

PMeOx$_{48}$-*b*-PiPrOx$_{39}$-phthalimide. Methyl *p*-toluenesulfonate (17.8 µL, 0.1 mmol, 1 eq), anhydrous acetonitrile (2.4 mL) and 2-methyl-2-oxazoline (0.5 mL, 5.9 mmol, 50 eq) were transferred under nitrogen to an oven-dried greaseless Schlenk flask equipped with magnetic stirrer. The flask was then sealed and the reaction mixture heated to 80 °C for 7.5 h. A 0.3 mL aliquot was removed from the reaction mixture for molecular weight analysis of the PMeOx block, then 2-isopropyl-2-oxazoline (0.6 mL, 5.1 mmol, 50 eq) added, the

flask sealed and the reaction mixture stirred at 80 °C for a further 16 h. To quench the reaction potassium phthalimide (0.150 g, 0.81 mmol, 8 eq) was added and the reaction mixture stirred at 70 °C for 14 h. Solids were removed by filtration and volatiles removed under vacuum. The resulting block copolymer was then precipitated directly into diethyl ether, followed by two subsequent precipitations from CH_2Cl_2 into diethyl ether. The resulting colourless solid was used without further purification. Yield = 0.7 g (65%)
^{1}H NMR (400 MHz, D_2O) δ: 7.96 – 7.82 (m, Ar, 4H), 4.00 – 3.87 (s, CONC*H*$_2$, 2H), 4.21 – 3.08 (m, NC*H*$_2$C*H*$_2$, 352H), 3.10 – 2.60 (d, CH_3C*H*CH_3, 39H), 2.26 – 1.91 (m, COC*H*$_3$, 144H), 1.56 – 0.67 (m, 2C*H*$_3$, 234H).
SEC (RI, DMAc): M_n ($Đ_m$) = 16,700 Da (1.10)

PMeOx$_{48}$-*b*-PiPrOx$_{39}$-NH$_2$. In a 50 mL round-bottom flask fitted with magnetic stirrer PMeOx$_{48}$-*b*-PiPrOx$_{39}$-phthalimide (0.7 g, 0.08 mmol, 1 eq) was dissolved in ethanol (25 mL) followed by the addition of hydrazine monohydrate (47 µL, 0.86 mmol, 11 eq). The reaction mixture was then heated to reflux for 22 h, cooled to room temperature and filtered. Subsequently, 5 M HCl (aq) was added to adjust the pH value of the solution to 2 and the resulting precipitate removed by filtration. Volatiles were then removed under vacuum and the residue dissolved in water. Saturated NaOH (aq) was added to increase the pH value of the solution to 10. This solution was transferred to 12-14 kDa MWCO dialysis tubing (Cellu-Sep T3), dialysed against water for 3 d and isolated by freeze-drying. The organic layer was dried over $MgSO_4$ and concentrated under vacuum. P*i*PrOx$_{40}$-NH$_2$ was isolated by precipitation into petroleum benzine. Yield = 220 mg (31%)
^{1}H NMR (400 MHz, D_2O) δ: 4.21 – 3.08 (m, NC*H*$_2$C*H*$_2$, 352H), 3.10 – 2.60 (d, CH_3C*H*CH_3, 39H), 2.26 – 1.91 (m, COC*H*$_3$, 144H), 1.56 – 0.67 (m, 2C*H*$_3$, 234H).
SEC (RI, DMAc): M_n ($Đ_m$) = 18,200 Da (1.09)

PMeOx$_{48}$-*b*-PiPrOx$_{39}$-Cy5. In an oven-dried greaseless Schlenk flask, PMeOx$_{48}$-*b*-PiPrOx$_{40}$-NH$_2$ (17 mg, 0.001 mmol, 1 eq), Cy5-NHS (7 mg, 0.006 mmol, 5 eq) and triethylamine (50 µL, 0.359 mmol, 293 eq) were dissolved in anhydrous *N*,*N*-dimethylformamide (1.0 mL). The reaction mixture was stirred under nitrogen in absence of light at room temperature for 14 h. Volatiles were removed under vacuum and the residue was re-dissolved in 2.0 mL of $CHCl_3$. Excess Cy5 was removed by preparative size exclusion column chromatography (Bio-BeadsTM S-X1, 600 - 14000 MW exclusion range) and P*i*PrOx$_{40}$-Cy5 isolated as a blue solid by freeze-drying. Yield = 12 mg (65%).
^{1}H NMR (400 MHz, D_2O) δ: 8.16 – 7.93 (br, C*H*CHC*H*, 2H), 7.68 – 7.20 (m, ArH, 8H), 6.71 – 6.48 (br, CHC*H*CH, H), 6.36 – 6.16 (br, 2CC*H*CH, 2H), 4.56 – 4.37 (br, NHC*H*$_2$CH$_2$N, 2H), 4.21 – 3.08 (m, NC*H*$_2$C*H*$_2$, 350H), 3.10 – 2.60 (d, CH_3C*H*CH_3, 39H), 2.26 – 1.91 (m, COC*H*$_3$, 144H), 1.74 – 1.58 (s, 4C*H*$_3$, 12H), 1.56 – 0.67 (m, 2C*H*$_3$, 234H).
SEC (RI, DMAc): M_n ($Đ_m$) = 17,200 Da (1.08)

Self-Assembly Procedures

Spontaneous nucleation method. PMeOx-*b*-P*i*PrOx block copolymers ($PMeOx_{25}$-*b*-$PiPrOx_{50}$ 1 mg/mL, $PMeOx_{48}$-*b*-$PiPrOx_{47}$ 2 mg/mL, $PMeOx_{96}$-*b*-$PiPrOx_{49}$ 4 mg/mL) were dissolved in Milli-Q water and filtered through a 0.45 µm nylon syringe filter. The polymer solutions were then heated in a sealed vial to 70 °C before cooling to 23 °C.

Nanoparticle sonication. Seed micelles were prepared by ultrasonication of micelles prepared by spontaneous nucleation (annealed at 70 °C for 1 week and used immediately). The ultrasonic processor was operated at 20% amplitude using an on:off pulse sequence of 2 s:2 s and samples cooled with an ice bath. A total sonication time of 40 minutes was used to prepare seeds from each polymer. Summary of nanoparticle length analysis is shown in Table S1 and histograms shown in Figure S10.

Seeded growth $PMeOx_{48}$-*b*-$PiPrOx_{47}$. Samples were prepared from an aqueous colloidal solution of $PMeOx_{48}$-*b*-$PiPrOx_{47}$ seeds (1 mg/mL) and an aqueous solution of $PMeOx_{48}$-*b*-$PiPrOx_{47}$ (1 mg/mL). Solutions were prepared as described in σ corresponds to standard deviation of particle length.
Table S2 and heated to 60 °C for 16 h before cooling to 23 °C. Summary of nanoparticle length analysis is shown in σ corresponds to standard deviation of particle length.
Table S2 and histograms shown in Figure S13.

Nanorod kinetic stability. To study the kinetic stability of POx nanorods, a 1 mg/mL D_2O solution of nanorods (L_n = 275 nm, L_w/L_n = 1.08) was prepared by annealing a mixture of 1.6 mL of $PMeOx_{48}$-*b*-$PiPrOx_{47}$ seeds (1 mg/ml in D_2O) and 6.4 mL of $PMeOx_{48}$-*b*-$PiPrOx_{47}$ unimer (1 mg/mL in D_2O) at 60 °C for 16 h. This solution was split into two aliquots and which were stored at either 23 or 37 °C. Nanorod stability was monitored by ^1H NMR spectroscopy and TEM.

Cy5-labelled $PMeOx_{48}$-*b*-$PiPrOx_{47}$ nanorods. Samples were prepared from an aqueous colloidal solution of $PMeOx_{48}$-*b*-$PiPrOx_{47}$ seeds (1 mg/mL) and aqueous solutions of $PMeOx_{48}$-*b*-$PiPrOx_{47}$ (1 mg/mL) and $PiPrOx_{40}$-Cy5 (1 mg/mL). Solutions were prepared as described in Table S3 and S4 and heated to 60 °C for 16 h before cooling to 23 °C. Summary of nanoparticle length analysis is shown in σ corresponds to standard deviation of particle length.
Table S2 and histograms shown in Figure S25.

Cy5 labelled nanorods used in the murine ex vivo biodistribution assays were concentrated to 10 mg/mL by reducing the volume of the nanorod solutions ten-fold using a centrifugal concentrator with a MWCO of 30 kDa. TEM analysis of these samples was carried out after concentration by centrifugal filtration, with samples diluted from 10 mg/mL to 1 mg/mL prior to drop casting.

Biology

Human blood immune cell association assay. The method described here is based closely on those described by Caruso, Kent and coworkers in suplimentary reference [2]. Human whole blood was taken via venupuncture from a single donor and stored in sodium heparin. Plasma-stripped blood was obtained by centrifugal washing of whole blood 4x with phosphate buffered saline (PBS). 90-100 µl of whole and plasma-stripped blood was added to FACS tubes and allowed to equilibrate at 37˚C for 10 min prior to addition of Cy5-labelled POx nanorods or free polymer (stock concentration: 1 mg/ml in water) to a final concentration of either 10 µg/ml. Samples were incubated in the dark at 37˚C (95% humidity, 5% CO_2) for 1 h. All samples were then transferred to ice for further processing: first, red blood cell lysis was carried out utilising BD PharmLyse, with samples then incubated with appropriate antibodies for 30 min. Immune cell types identified by this assay include: CD66b+ neutrophils, CD14+ monocytes, CD19+ B cells, CD3+ T cells, CD56+ natural killer (NK) cells, and Lin1- HLA-DR+ dendritic cells (DCs); all antibodies were purchased from BD Biosciences. Samples were then washed in FACS wash buffer (2 mM EDTA, 0.5% bovine serum albumin in PBS) before cells were fixed with 10% formaldehyde. Characterisation was carried out on a BD LSRFortessa flow cytometer at medium speed, 5 decades and the FSC threshold set at 5,000. Data were analysed in FlowJo, graphed in GraphPad Prism and represent n = 2 independent experiments and n = 3 technical repeats. Raw cell counts are shown in the Supporting Schemes and Tables section.

Ex Vivo Biodistribution. Mice 8 weeks of age were intravenously injected with 100 µl of 10 mg/ml nanorod solution and 24 h post injection euthanized by CO_2. The heart, brain, stomach, liver, spleen, kidney, lung, muscle and blood were collected and wet-weighed. The organs were placed on a petri dish and imaged using an IVIS Lumina II imaging system (Caliper Life Sciences). All imaging parameters were kept constant and were as follows: Bin, (M)4; FOV: 12.5; f2; 2 s. The total radiant efficiency (p/s)/(µW/cm^2) of the organs as well as the standards (injected samples) were measured and background corrected. Based on the weight of the individual organs and total radiant efficiency, the data were then converted to %ID tissue g^{-1}. For statistical analysis with a group size of n=3 per nanorod sample, data were compared by two-way ANOVA with Tukey multiple comparisons post hoc test. Statistical significance was accepted at $p<0.05$. Ethical approval for animal experiments were granted from the Alfred Research Alliance Animal Ethics Committee under E/1625/2016/M.

Supporting Schemes and Tables

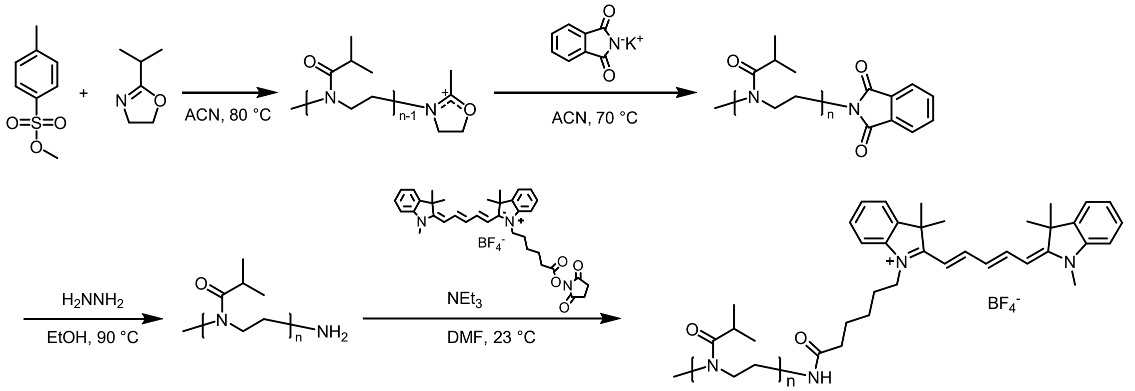

Scheme S1. Synthesis of P*i*PrOx-Cy5 via P*i*PrOx-phthalimide and P*i*PrOx-NH_2.

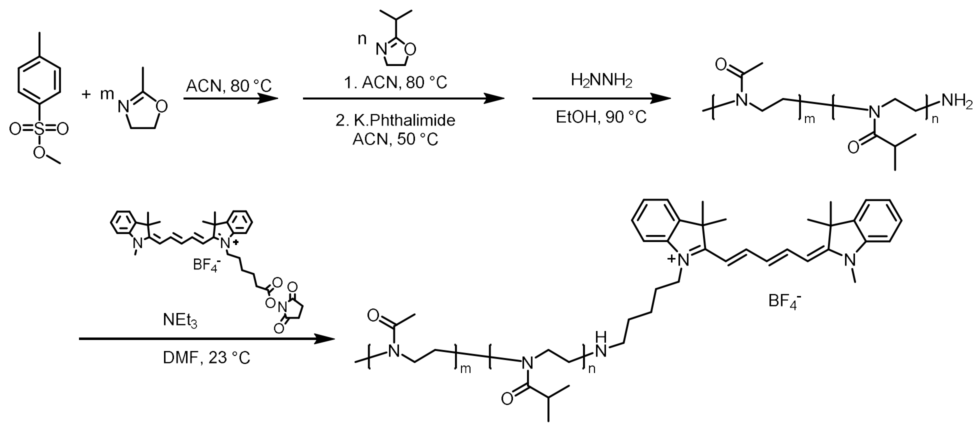

Scheme S2. Synthesis of PMeOx-*b*-P*i*PrOx-Cy5 via PMeOx-*b*-P*i*PrOx-phthalimide and PMeOx-*b*-P*i*PrOx-NH_2.

Table S1. Contour length analysis by TEM of $PMeOx_m$-*b*-P*i*$PrOx_n$ seed micelles prepared by ultrasonication.

Polymer	L_n (nm)	L_w (nm)	L_w/L_n	σ (nm)
$PMeOx_{24}$-*b*-P*i*$PrOx_{50}$	65	65	1.17	23
$PMeOx_{48}$-*b*-P*i*$PrOx_{47}$	45	53	1.17	18

σ corresponds to standard deviation of particle length.

Table S2. Sample preparation conditions and contour length analysis by TEM for $PMeO_{48}$-*b*-P*i*$PrOx_{47}$ nanorods prepared by seeded growth.

$\frac{mass_{unimer}}{mass_{seeds}}$	Vol. Seeds (mL)	Vol. Unimer (mL)	Vol. Water (mL)	L_n (nm)	σ (nm)	L_w/L_n	L_n/eq (nm/eq)
Seeds	2.000	-	-	123	47	1.15	123
1	1.000	1.000	-	263	74	1.08	132
2	0.667	1.333	-	324	105	1.10	108
3	0.500	1.500	-	441	135	1.09	110
4	0.400	1.600	-	635	132	1.04	127

Table S3. Sample preparation conditions and contour length analysis by TEM for Cy5-labelled PMeOx$_{48}$-*b*-P*i*PrOx$_{47}$ nanorods used in immune cell association assay.

Sample	Vol. Seeds (mL)	Vol. BCP Unimer (mL)	Vol. P*i*PrOx$_{40}$-Cy5 (mL)	L_n (nm)	σ (nm)	L_w/L_n
Short 1	1.980	-	0.020	63	25	1.16
Short 2	1.980	-	0.020	69	30	1.19
Medium 1	0.571	1.409	0.020	216	57	1.07
Medium 2	0.571	1.409	0.020	215	65	1.09
Long 1	0.235	1.745	0.020	467	140	1.09
Long 2	0.235	1.745	0.020	434	138	1.10

Table S4. Sample preparation conditions and contour length analysis by TEM for Cy5-labelled PMeOx$_{48}$-*b*-P*i*PrOx$_{47}$ used in murine ex vivo biodistribution study.

Sample	Vol. Seeds (mL)	Vol. BCP Unimer (mL)	Vol. P*i*PrOx$_{40}$-Cy5 (mL)	L_n (nm)	σ (nm)	L_w/L_n
Short 3	10.395	-	0.105	69	30	1.18
Medium 3	5.250	5.145	0.105	146	39	1.07
Long 3	2.625	7.770	0.105	284	67	1.05

Table S5. Raw cell counts for flow cytometry analysis of immune cell assocation in whole blood.

	Control		Short		Medium		Long		PMeOx-b-PiPrOx-Cy5
Repeat	1	2	1	2	1	2	1	2	
Total count	5.19E+05	5.27E+05	355166	8.85E+05	5.22E+05	264155	4.36E+05	6.42E+05	6.27E+05
Granulocytes	16597	18961	20090	18659	23618	17216	21463	12255	8188
Single cells	14832	17439	18899	17412	21696	16217	20087	16562	6189
Neutrophils	14528	17117	18702	11126	21364	15971	19908	10447	5288
↘ Cy5+	436	38	36	300	494	42	74	304	5194
Lymphocytes	57487	70797	65228	102071	57404	45130	59077	99376	48244
Single cells	55987	67402	62001	99684	56134	42977	55510	97405	45297
T cells	37210	53421	49176	74477	36092	33310	43881	74121	32400
↘ Cy5+	8	6	21	16	127	14	19	151	22868
CD14-, CD3-	18427	13362	12207	24304	19617	8906	11006	22119	9413
B cells	5413	5575	5593	9665	4977	3302	3712	9327	3496
↘ Cy5+	6	7	270	878	78	13	20	39	3129
NK cells	3399	4482	3829	7053	3782	2484	3751	6056	3618
↘ Cy5+	9	2	1	4	32	3	2	19	2776
CD56-, CD19-	9423	3096	2634	7126	10626	2960	3353	6437	2197
DCs	235	294	257	546	239	228	256	463	37
↘ Cy5+	1	2	1	8	1	1	4	3	29
Monocytes	4105	2566	2253	5760	4284	1676	2093	6401	2522
Single cells	3947	2380	2108	5153	4089	1552	1967	5750	2272
CD14+ monocytes	2987	1471	1190	3997	3080	926	1271	4645	1479
↘ Cy5+	52	17	12	31	81	5	14	56	1475

Table S6. Raw cell counts for flow cytometry analysis of immune cell assocation in plasma-stripped blood.

	Control		Short		Medium		Long		PMeOx-*b*-P*i*PrOx-Cy5
Repeat	*1*	*2*	*1*	*2*	*1*	*2*	*1*	*2*	
**Total count**	1.90E+05	110998	119522	2.52E+05	200491	103523	123464	257393	179515
**Granulocytes**	12537	8070	9152	17934	9566	11138	8937	10513	3340
Single cells	11572	7537	8694	17158	8799	10723	8454	9852	2686
Neutrophils	11292	7483	8571	16650	8523	10644	8386	9549	2542
↘ Cy5+	515	50	45	469	419	66	73	406	2524
**Lymphocytes**	36283	24992	26887	56098	39248	24378	24771	48428	28428
Single cells	35739	24603	26466	55575	38666	24093	24253	47987	27572
T cells	24169	19610	20208	41813	26771	18146	19074	36896	19095
↘ Cy5+	4	28	30	13	56	165	40	63	18417
CD14-, CD3-	11326	4787	5761	13122	11649	5515	4952	10501	4680
B cells	2750	1694	1503	4030	2817	1236	2039	3101	1779
↘ Cy5+	6	2	3	4	16	21	7	4	1699
NK cells	2823	1626	1770	4790	2853	1672	1871	3851	1717
↘ Cy5+	13	6	5	1	33	24	8	8	1686
CD56-, CD19-	5570	1343	2382	3980	5779	2509	907	3263	1150
DCs	250	246	236	580	246	203	165	491	41
↘ Cy5+	3	0	1	4	5	8	5	10	39
**Monocytes**	3961	1250	1325	5144	3250	1352	1599	3287	1110
Single cells	3860	1211	1277	4667	3119	1318	1537	2798	930
CD14+ monocytes	2927	687	777	3634	2288	921	1101	1989	489
↘ Cy5+	17	2	3	29	23	8	1	29	489

Supporting Figures

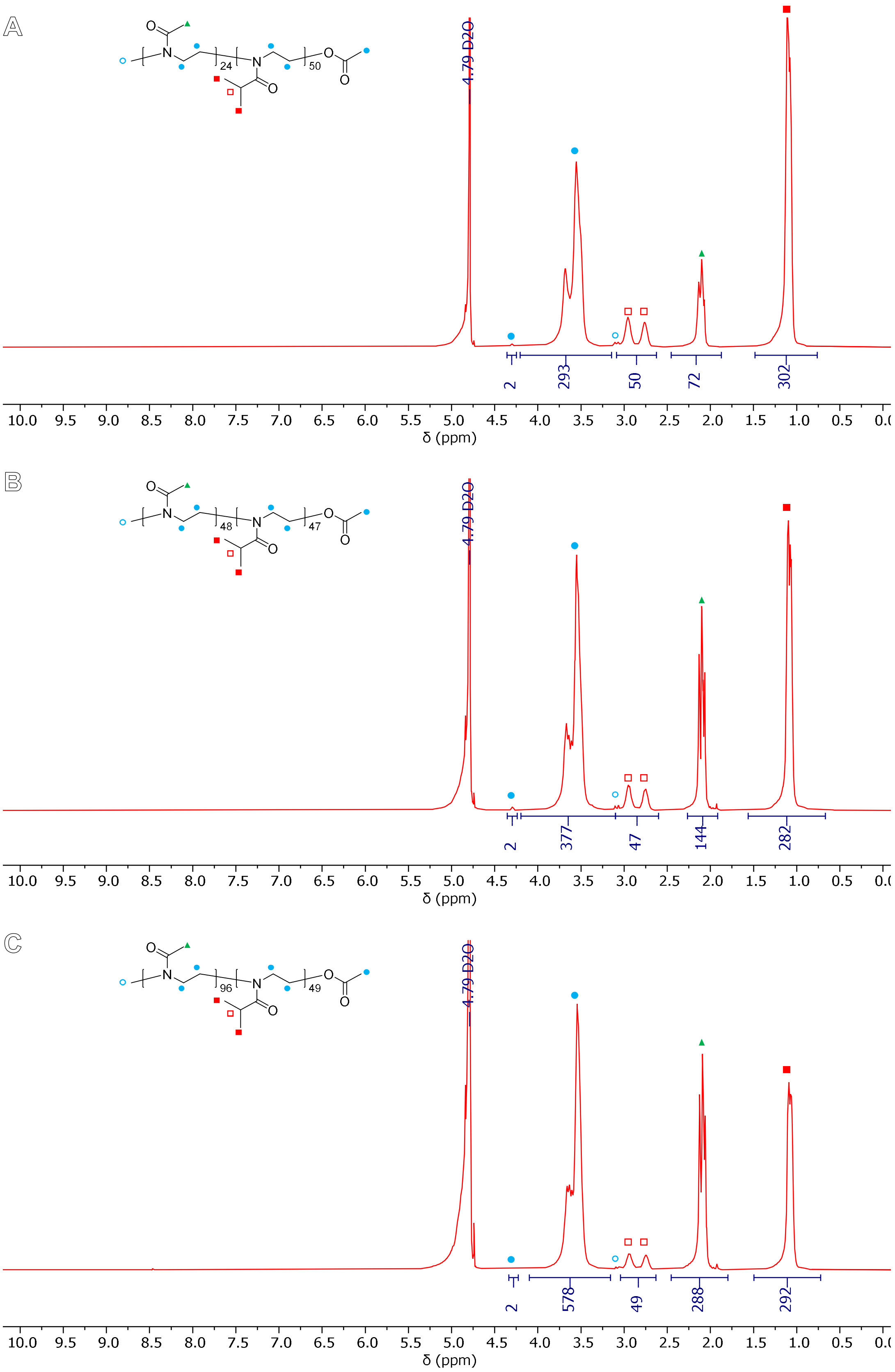

Figure S1. ^{1}H NMR (D_2O, 400 MHz) spectra of PMeOx$_m$-*b*-P*i*PrOx$_n$ block copolymers. A) PMeOx$_{24}$-*b*-P*i*PrOx$_{50}$, B) PMeOx$_{48}$-*b*-P*i*PrOx$_{47}$, C) PMeOx$_{96}$-*b*-P*i*PrOx$_{49}$.

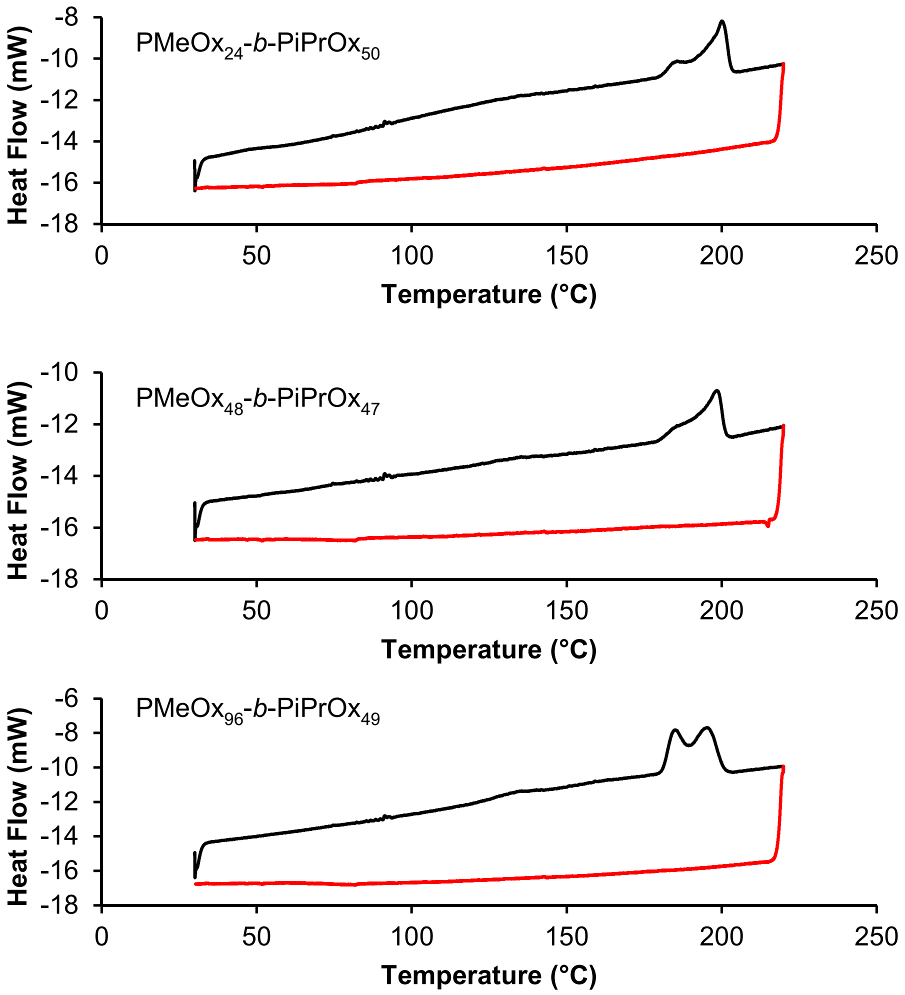

Figure S2. DSC analysis of $PMeOx_m$-*b*-P*i*$PrOx_n$ block copolymers (heating scan black, cooling scan red, endotherms up, scan rate 10 °C/min). Prior to DSC analysis polymers were, under nitrogen, first heated to 210 °C for 10 min to erase thermal history, then annealed at 130 °C for 14 h to allow P*i*PrOx blocks to crystallise.

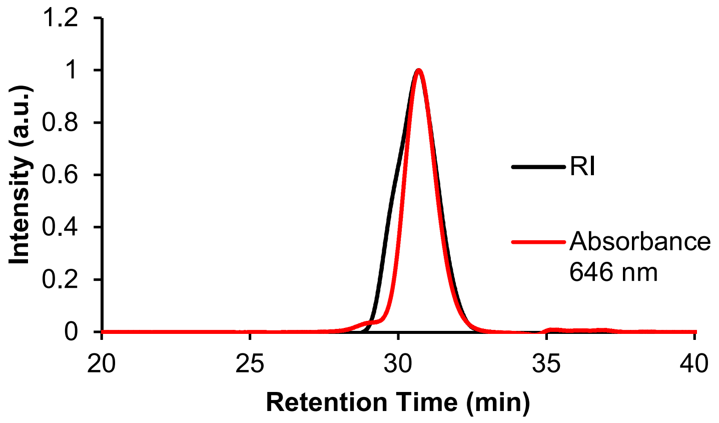

Figure S3. SEC chromatogram of P*i*$PrOx_{40}$-Cy5 (absorbance at 646 nm red and RI detector black, DMAc 0.03 wt% LiBr,1 mL/min).

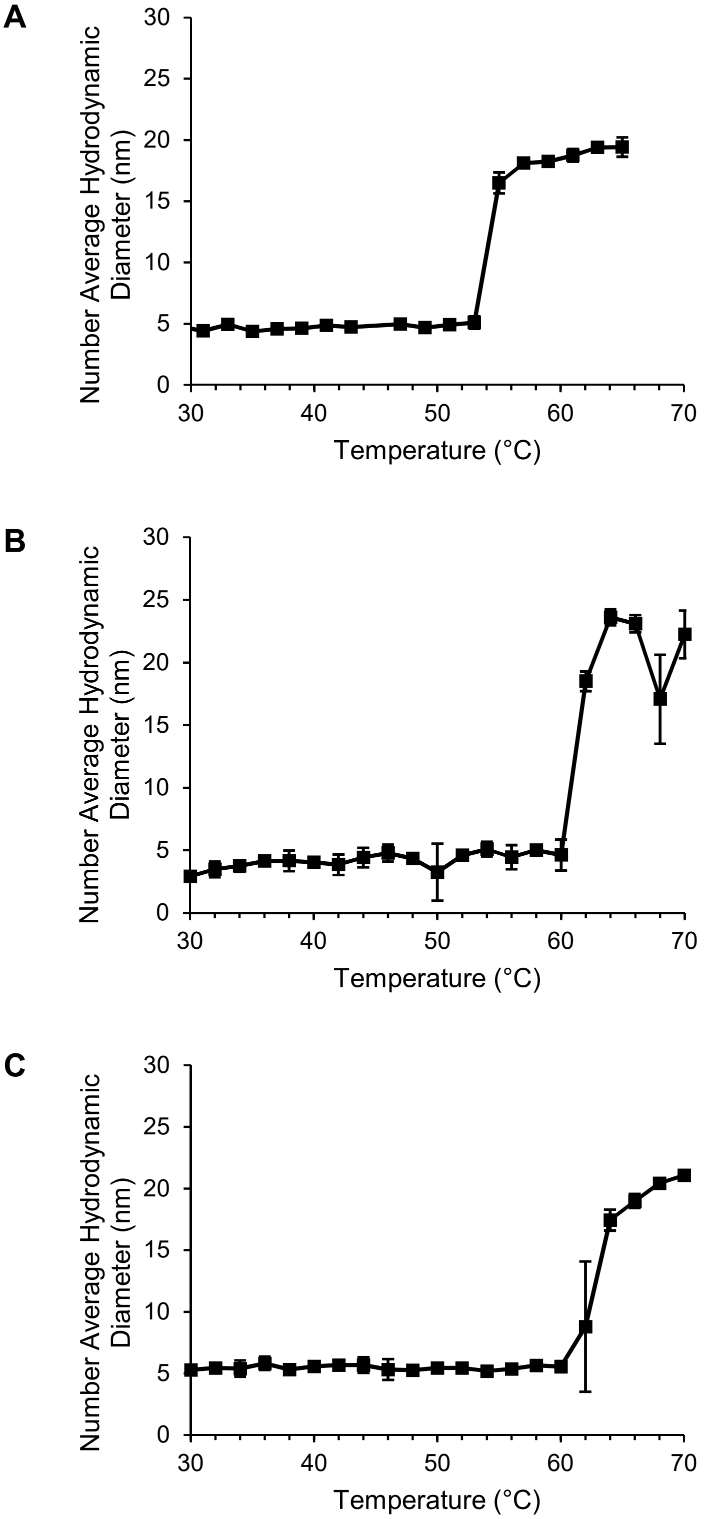

Figure S4. Cloud point temperature determination by DLS for PMeOx$_m$-*b*-P*i*PrOx$_n$ block copolymers (10 mg/mL in MilliQ water). Y-axis reports mean hydrodynamic diameter from n = 3 measurements, error bars correspond to standard deviation. A) PMeOx$_{24}$-*b*-P*i*PrOx$_{50}$, B) PMeOx$_{48}$-*b*-P*i*PrOx$_{47}$, C) PMeOx$_{96}$-*b*-P*i*PrOx$_{49}$.

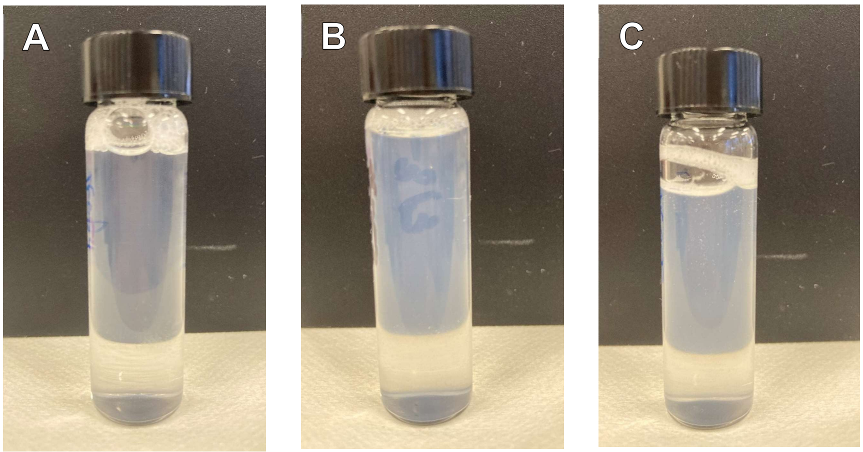

Figure S5. Photographs of aqueous PMeOx$_{m}$-*b*-P*i*PrOx$_{n}$ suspensions after annealing at 70 °C. A) PMeOx$_{24}$-*b*-P*i*PrOX$_{50}$ 1 mg/mL annealed at 70 °C for 24 h. B) PMeOx$_{48}$-*b*-P*i*PrOx$_{47}$ 2 mg/mL annealed at 70 °C for 24 h. C) PMeOx$_{96}$-*b*-P*i*PrOX$_{49}$ 4 mg/mL annealed at 70 °C for 1 week.

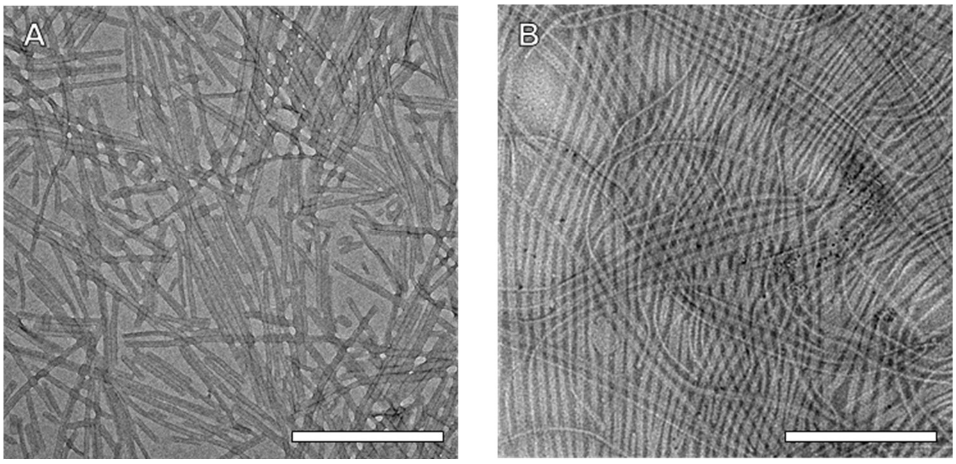

Figure S6. TEM images of PMeOx$_{m}$-*b*-P*i*PrOx$_{n}$ fibres prepared by spontaneous nucleation method. Polymer solutions were annealed at 70 °C for 1 week. Scale bar equal to 500 nm. A) PMeOx$_{24}$-*b*-P*i*PrOX$_{50}$. B) PMeOx$_{96}$-*b*-P*i*PrOx$_{49}$.

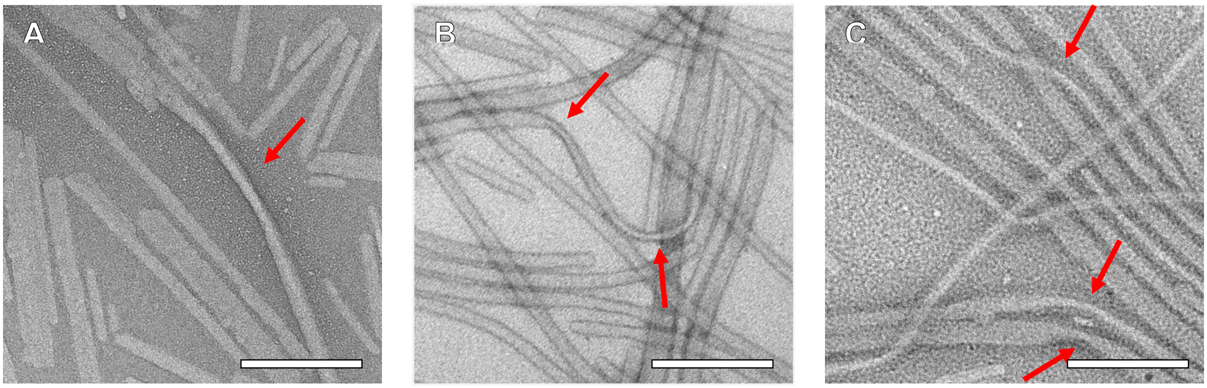

Figure S7. TEM images of PMeOx$_{24}$-*b*-P*i*PrOx$_{50}$ (A), PMeOx$_{48}$-*b*-P*i*PrOx$_{47}$ (B) and PMeOx$_{96}$-*b*-P*i*PrOx$_{49}$ (C) fibres prepared by spontaneous nucleation method. Twists in fibre long axis that indicate a non-circular cross-section are denoted by red arrows. Scale bars equal to 200 nm.

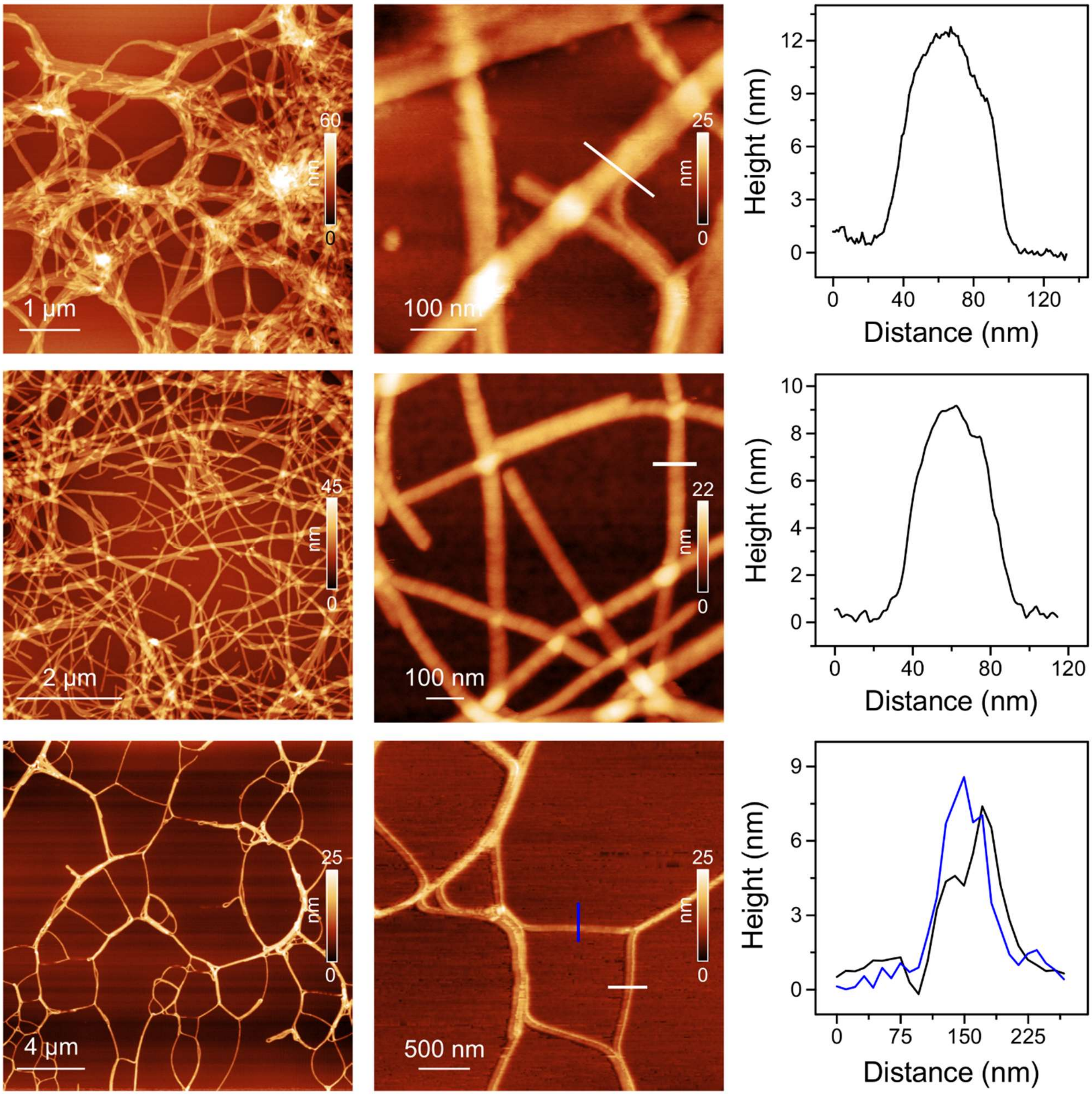

Figure S8. Topographic images (height channel) and height profiles of PMeOx$_m$-*b*-P*i*PrOx$_n$ NPs prepared by spontaneous nucleation method from AFM analysis. Polymer solutions were annealed at 70 °C for 1 week. Scale bar equal to 500 nm. Top) PMeOx$_{24}$-*b*-P*i*PrOX$_{50}$, middle) PMeOx$_{48}$-*b*-P*i*PrOx$_{47,}$ bottom) PMeOx$_{96}$-*b*-P*i*PrOx$_{49}$.

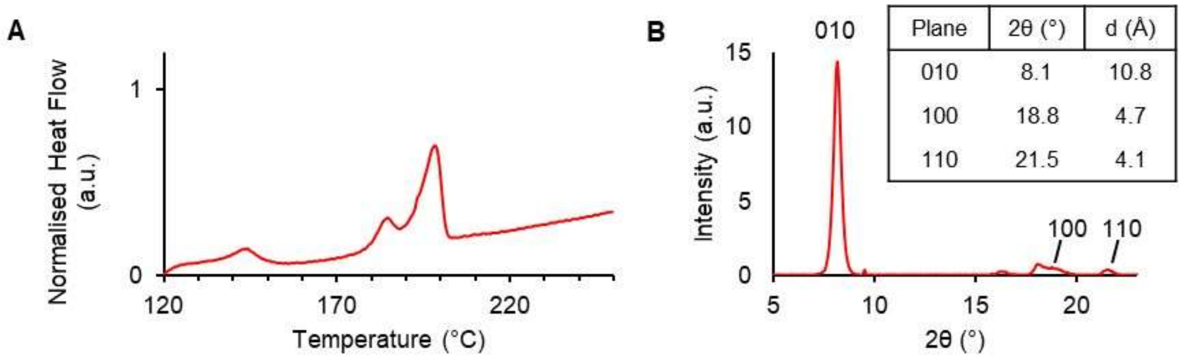

Figure S9. A) First DSC heating scan of freeze-dried PMeOx$_{48}$-*b*-P*i*PrOx$_{47}$ fibres (endotherms up, scan rate 10 °C/min). B) WAXS analysis of freeze-dried PMeOx$_{48}$-*b*-P*i*PrOx$_{47}$ fibres. Lattice planes assigned according to M. Litt et al, *J. Polym. Sci. Part A-2 Polym. Phys.* **1969**, *7*, 463–473.

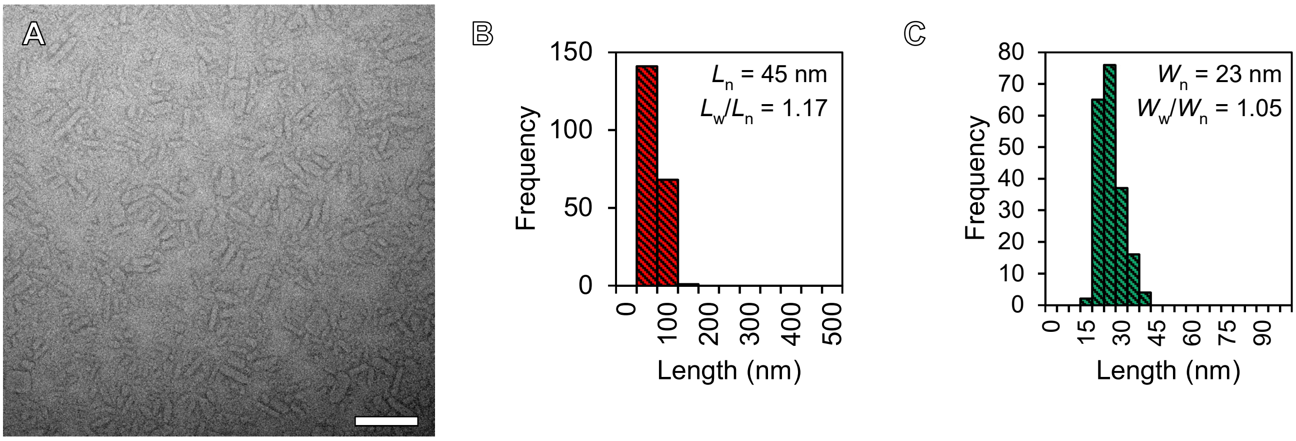

Figure S10. A) TEM image of PMeOx$_{48}$-*b*-P*i*PrOx$_{47}$ fibre fragments prepared by ultrasonication. Scale bar equal to 200 nm. B) Nanoparticle length histogram based on TEM analysis. C) Nanoparticle width histogram based on TEM analysis.

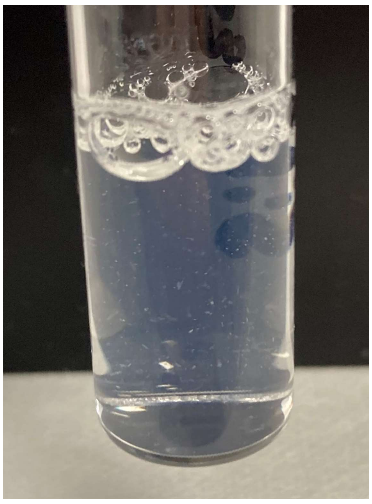

Figure S11. Photograph of PMeOx$_{48}$-*b*-P*i*PrOx$_{47}$ suspension (m_{unimer}/m_{seed} = 1, 1 mg/mL) after annealing at 70 °C for 16 h.

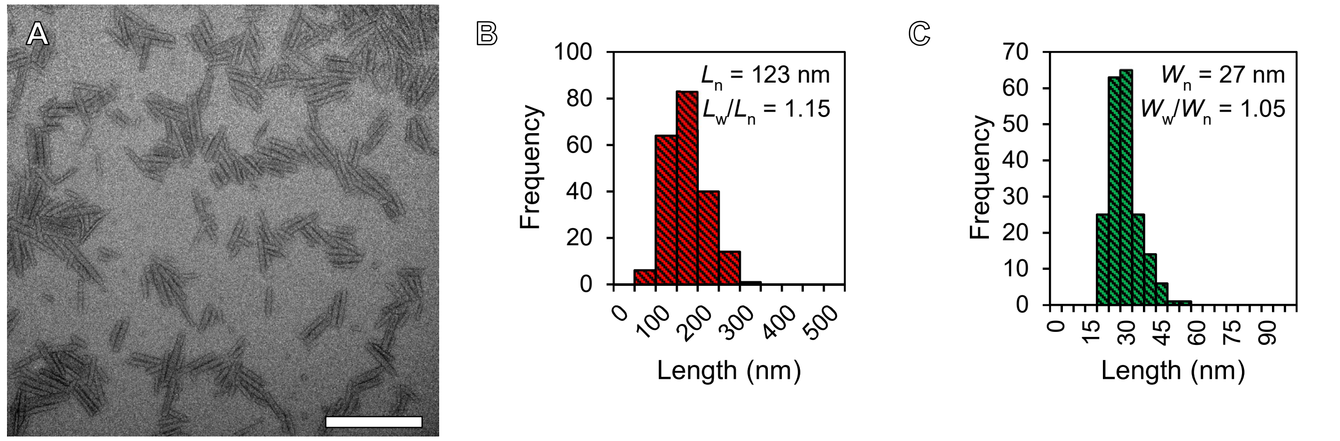

Figure S12. A) TEM images of PMeOx$_{48}$-*b*-P*i*PrOx$_{47}$ nanorods prepared by annealing (60 °C, 16 h) seed fragments directly after ultrasonication without the addition of additional unimer. Scale bar equal to 500 nm. B) Nanoparticle length histogram based on TEM analysis. C) Nanoparticle width histogram based on TEM analysis.

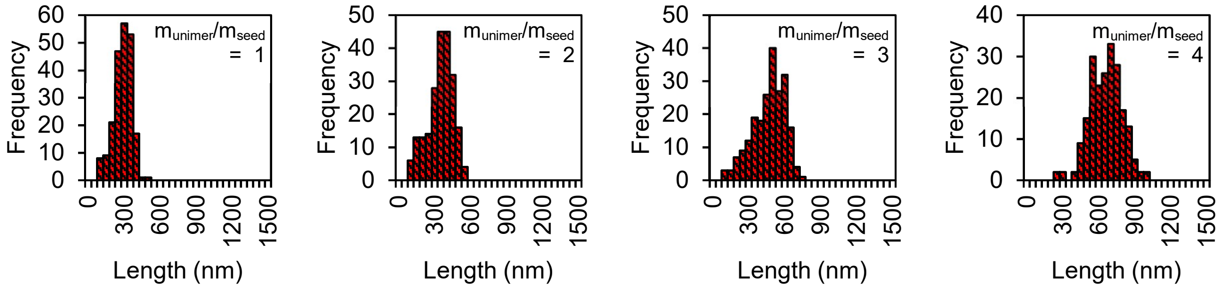

Figure S13. PMeOx$_{48}$-*b*-P*i*PrOx$_{47}$ nanoparticle length histograms based on TEM analysis.

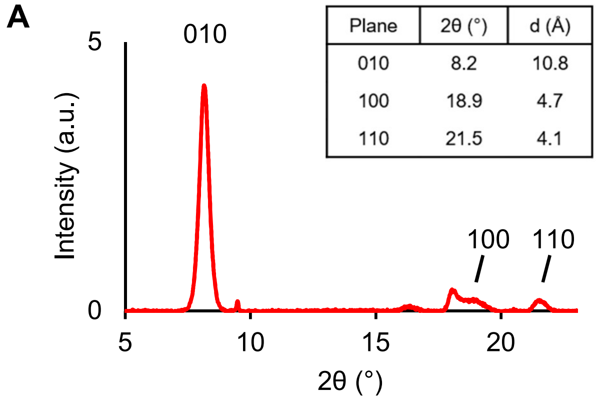

Figure S14. A) WAXS analysis of freeze-dried PMeOx$_{48}$-*b*-P*i*PrOx$_{47}$ nanorods prepared by seeded growth with a unimer to seed mass ratio of 4. Lattice planes assigned according to M. Litt et al, *J. Polym. Sci. Part A-2 Polym. Phys.* **1969**, *7*, 463–473.

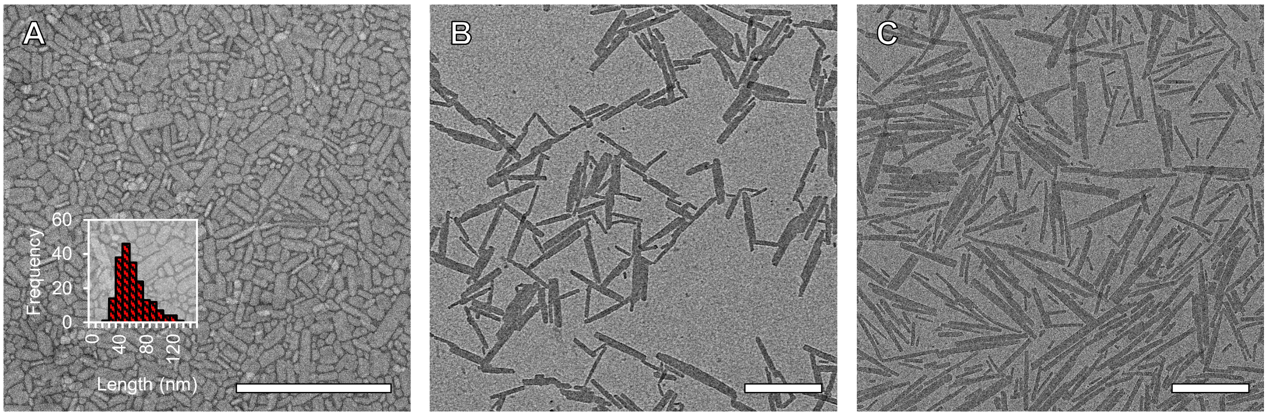

Figure S15. TEM images of $PMeOx_{24}$-*b*-P*i*$PrOx_{50}$ nanorods. A) Fibre fragments prepared by ultrasonication. B) Nanorods prepared by seeded growth at 60 °C (0.5 mg/mL) with a unimer to seed ratio of 2. C) Nanorods prepared by seeded growth at 60 °C (0.5 mg/mL) with a unimer to seed ratio of 4. Scale bars equal to 500 nm.

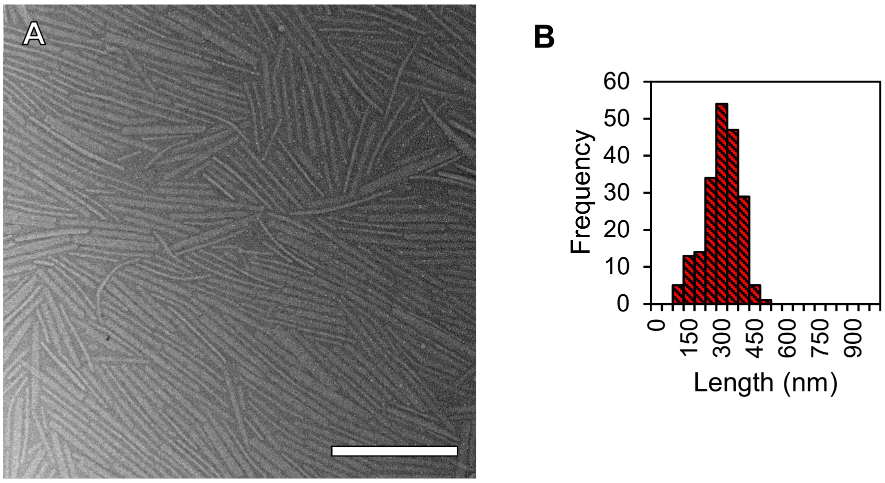

Figure S16. A) TEM image of $PMeOx_{48}$-*b*-P*i*$PrOx_{47}$ nanorods prepared for stability study, L_n = 275 nm, L_w/L_n = 1.08. Scale bars equal to 500 nm. B) Corresponding micelle length histogram.

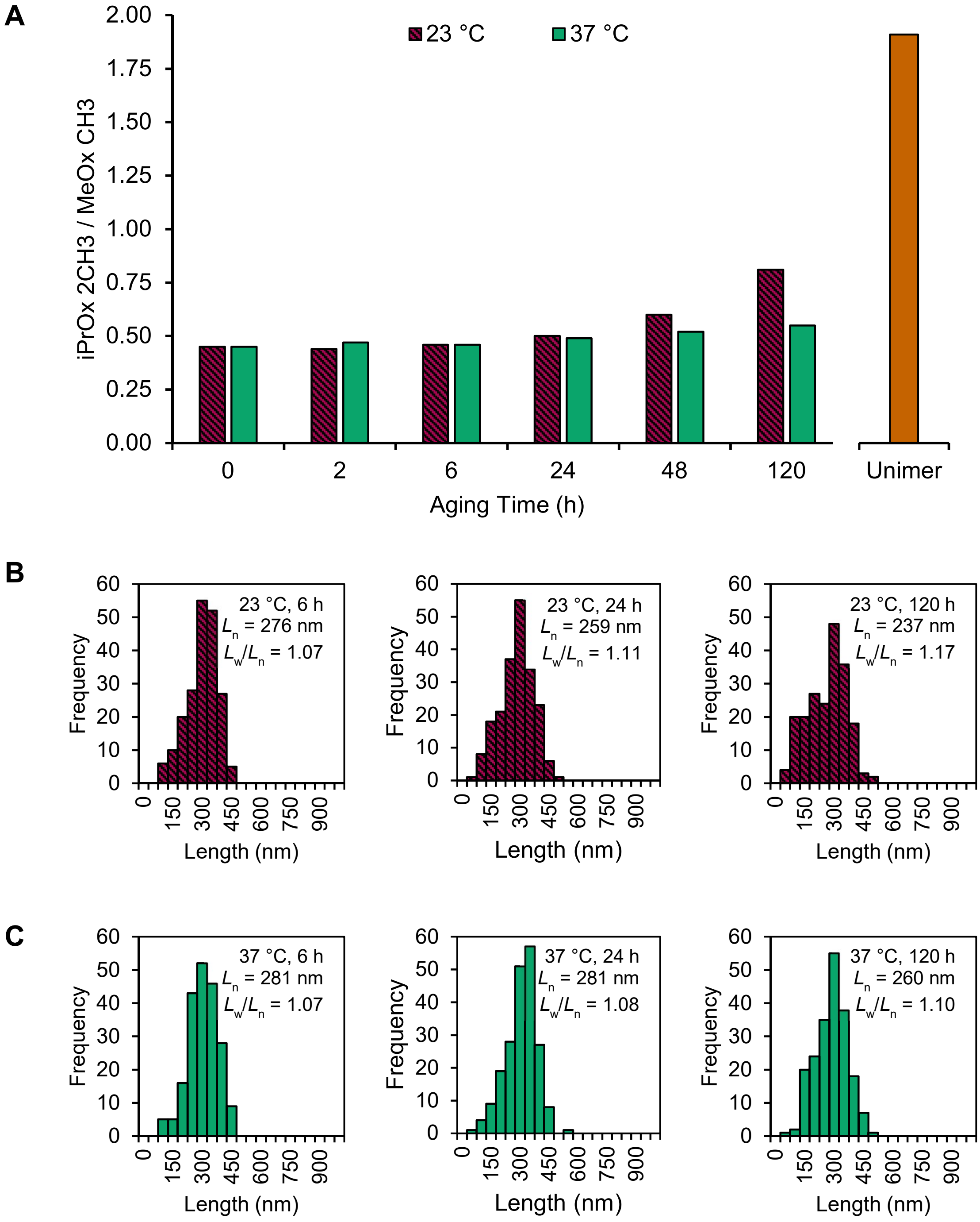

Figure S17. PMeOx$_{48}$-*b*-P*i*PrOx$_{47}$ nanorod kinetic stability study. A) Plot showing increase over time in the ratio of the ^{1}H resonance (400 MHz, D_2O) from the *i*PrOx 2C*H*$_3$ to that of the PMeOx C*H*$_3$ when a solution of PMeOx$_{48}$-*b*-P*i*PrOx$_{47}$ nanorods (L_n = 275 nm, L_w/L_n = 1.08) was aged in D_2O at 23 and 37 °C. For reference the ratio of these signals for a unimeric solution of PMeOx$_{48}$-*b*-P*i*PrOx$_{47}$ is also shown. B) Micelle length histograms based on TEM analysis for samples stored at 23 °C. C) Micelle length histograms based on TEM analysis for samples stored at 37 °C.

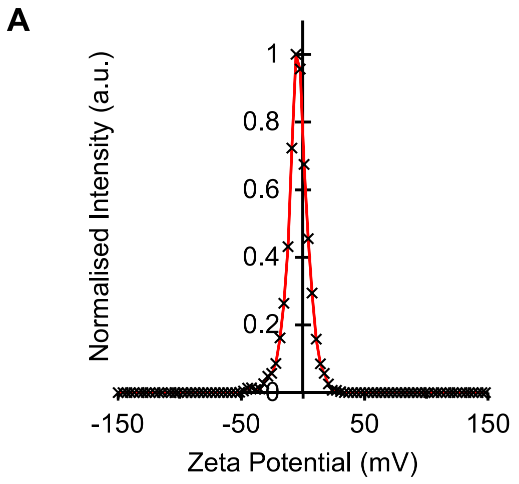

Figure S18. Zeta potential plots for (A) PMeOx48-*b*-P*i*PrOx47 nanorods prepared by seeded growth (m_{unimer}/m_{seed} = 4), and (B) Cy5-labelled PMeOx48-*b*-P*i*PrOx47 nanorods prepared by seeded growth (m_{unimer}/m_{seed} = 4, 1 wt% PiPrOx40-Cy5). Zeta potential measured at 1 mg/mL in Milli-Q water, plots represent the mean of 3 individual measurements.

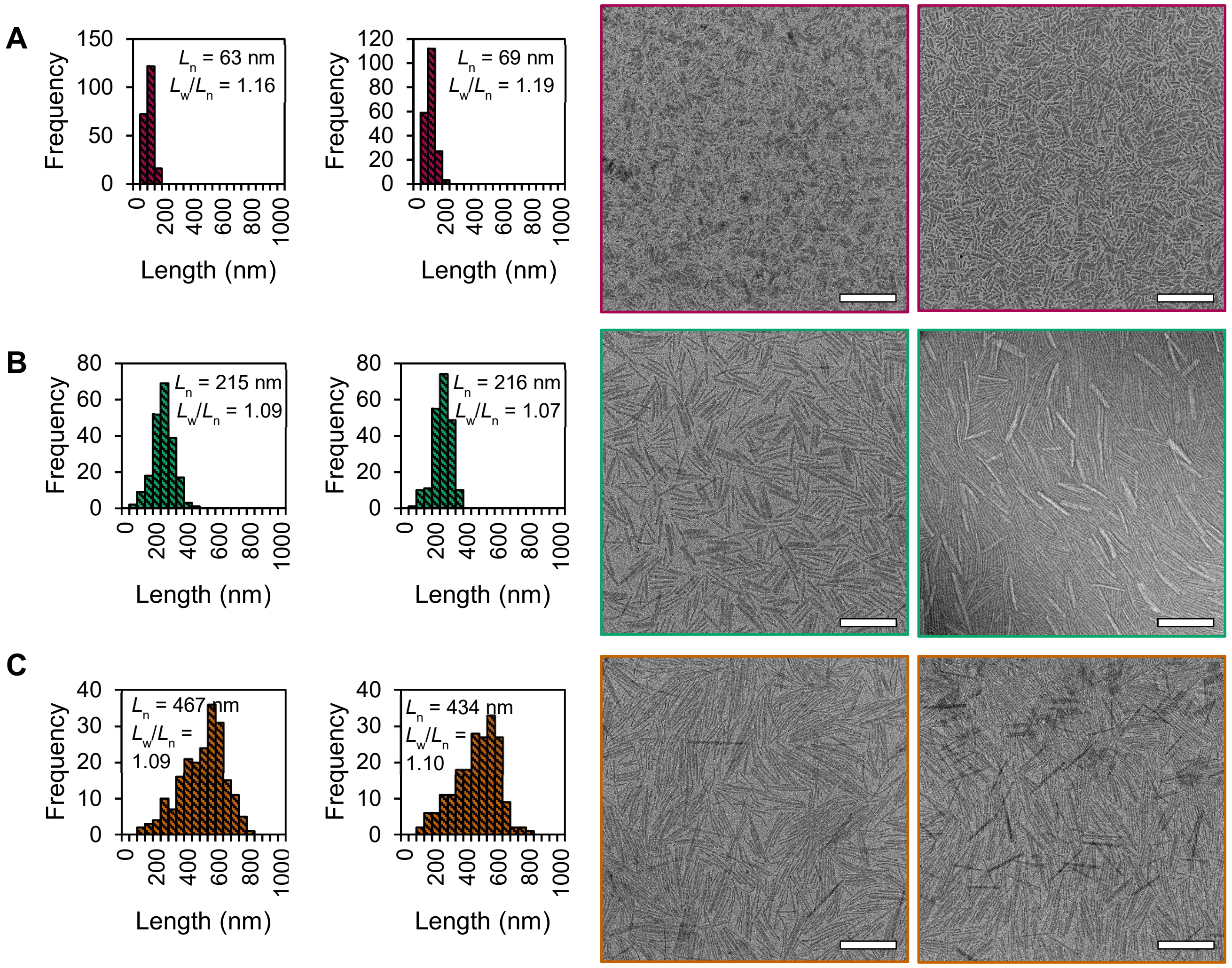

Figure S19. Micelle length histograms (left) based on TEM analysis and representative TEM image (right) for Cy5 labelled POx 'short' (A), 'medium' (B) and 'long' (C) nanorods used in immune cell association assay.

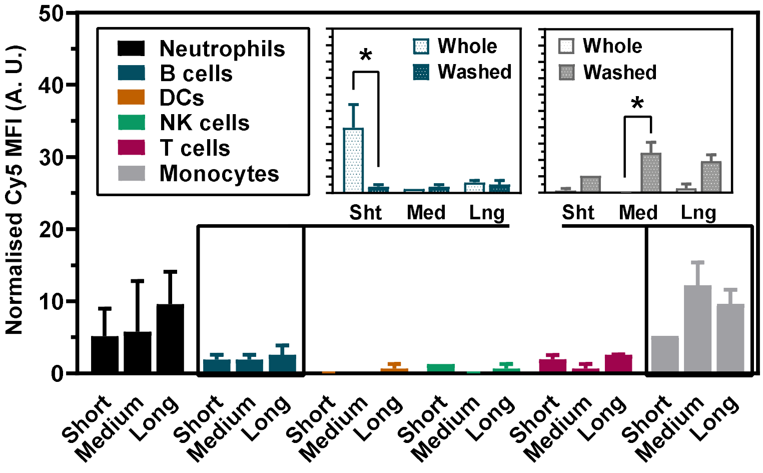

Figure S20. Normalised Cy5 mean fluorescence intensity (MFI) associated with different immune cell types in 'washed' (plasma-stripped) blood after incubation with POx-Cy5 nanorods for 1 h. Insets: comparative MFI of POx nanorods interacting with B cells (blue) and monocytes (grey) in the presence (whole) and absence (washed) of plasma. Error is standard error of mean (n = 2); * p < 0.05 by a two-way ANOVA with Sidak's correction for multiple comparisons.

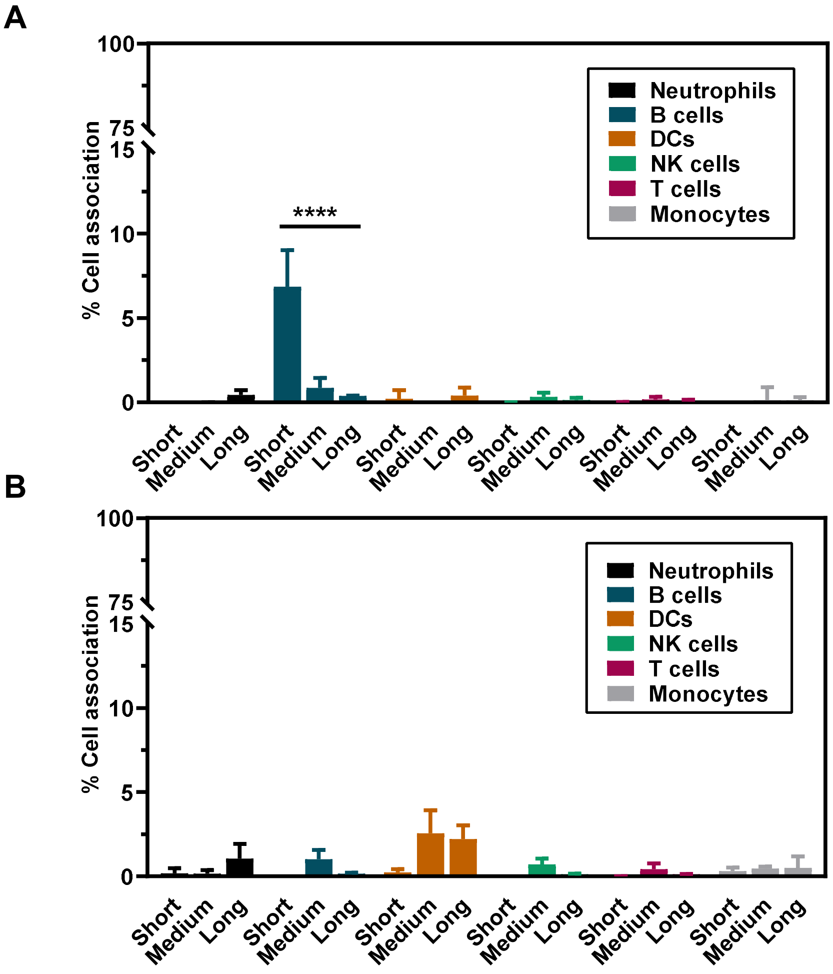

Figure S21. Immune cell association of POx-Cy5 nanorods after incubation for 1 h as a percentage of total cell population, in human (A) whole and (B) washed blood. Error is standard error of mean (n = 2); **** p < 0.0001 by a two-way ANOVA with Sidak's correction for multiple comparisons.

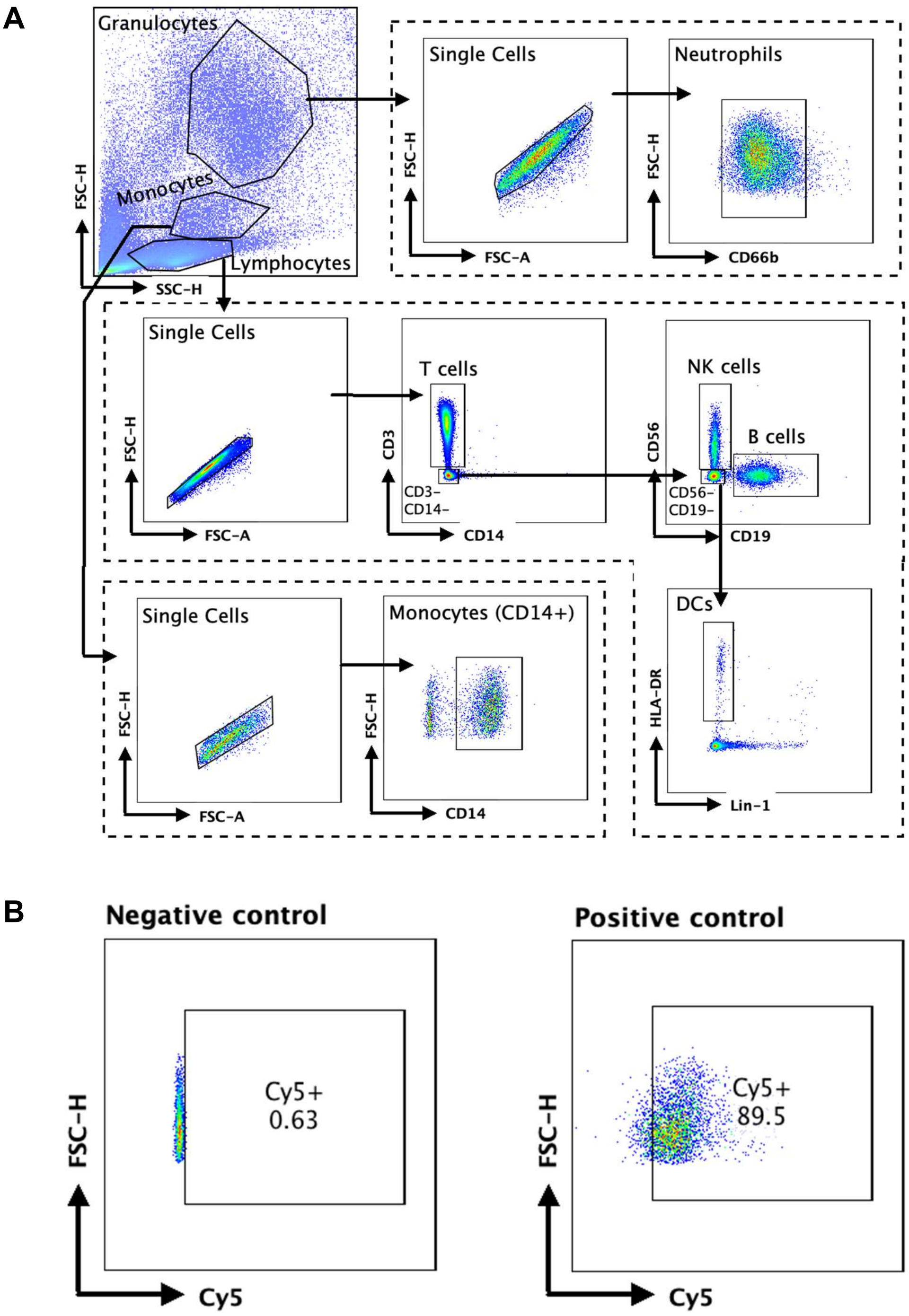

Figure S22. Gating strategy for flow cytometry analysis of Cy5-labelled POx nanorod localisation to immune cells in whole and washed blood. A) White blood cells were first identified and doublets excluded before specific cell types were assigned based on surface marker expression: CD66b+ neutrophils, CD3+ T cells, CD14+ monocytes, CD56+ natural killer (NK) cells, CD19+ B cells, and Lin-1- HLA-DR+ dendritic cells (DCs). B) Representative example of gating to identify POx-associated cells. Figure shows B cell negative control (cells only) and positive control (cells incubated with PMeOx$_{48}$-*b*-P*i*PrOx$_{40}$-Cy5).

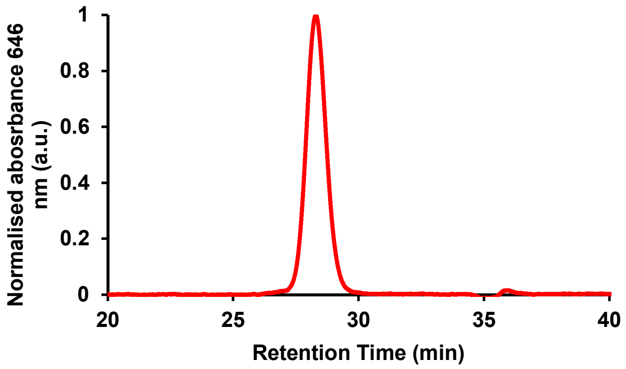

Figure S23. SEC chromatogram (absorbance at 646 nm, DMAc 0.03 wt% LiBr,1 mL/min) of PMeOx$_{48}$-*b*-P*i*PrOx$_{39}$-Cy5.

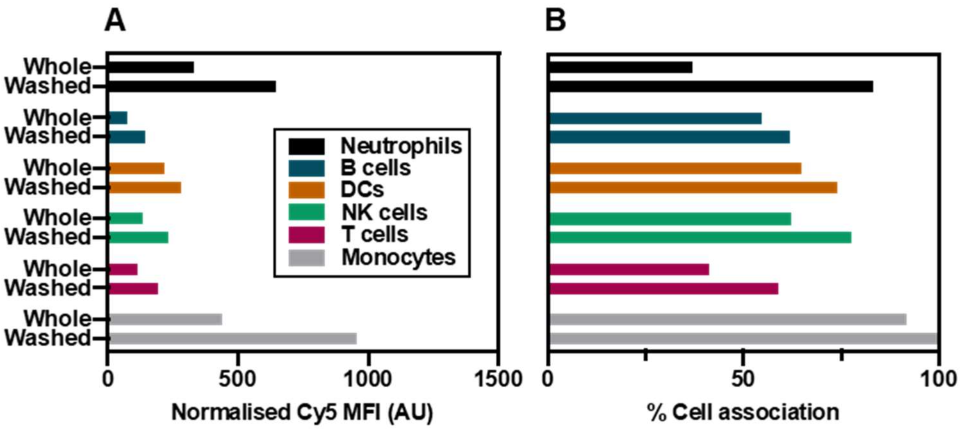

Figure S24. Immune cell association of PMeOx$_{48}$-*b*-P*i*PrOx$_{40}$-Cy5 after incubation for 1 h as (A) normalised Cy5 mean fluorescence intensity (MFI) and (B) percentage association within the total cell population in human whole and washed blood.

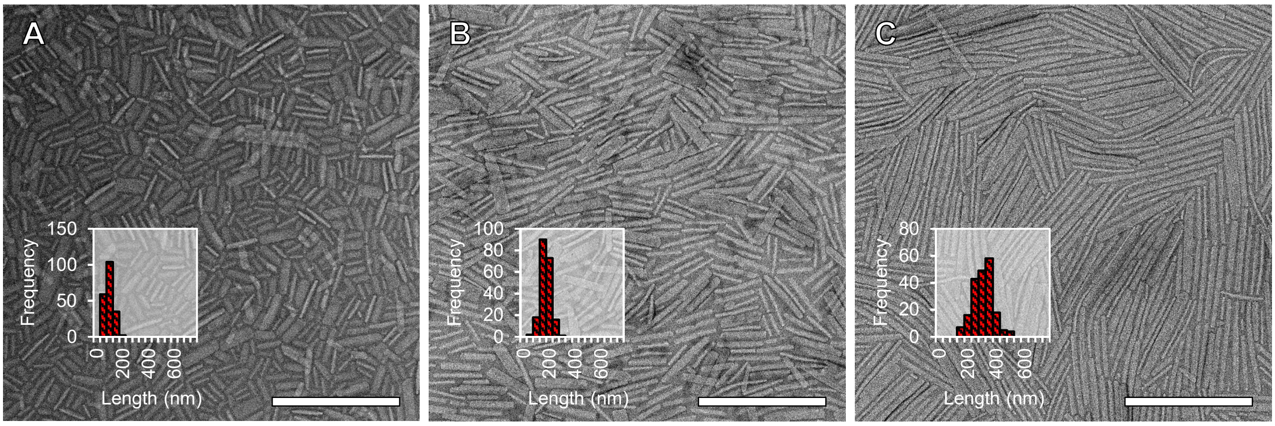

Figure S25. TEM images and length histograms based on TEM analysis for Cy5 labelled POx nanorods used in murine ex vivo biodistribution study. A) L_n = 69 nm, B) L_n = 146 nm, C) L_n = 284 nm. Scale bars equal to 500 nm. TEM analysis was carried out after concentration by centrifugal filtration with samples diluted from 10 mg/mL back to 1 mg/mL prior to drop casting.

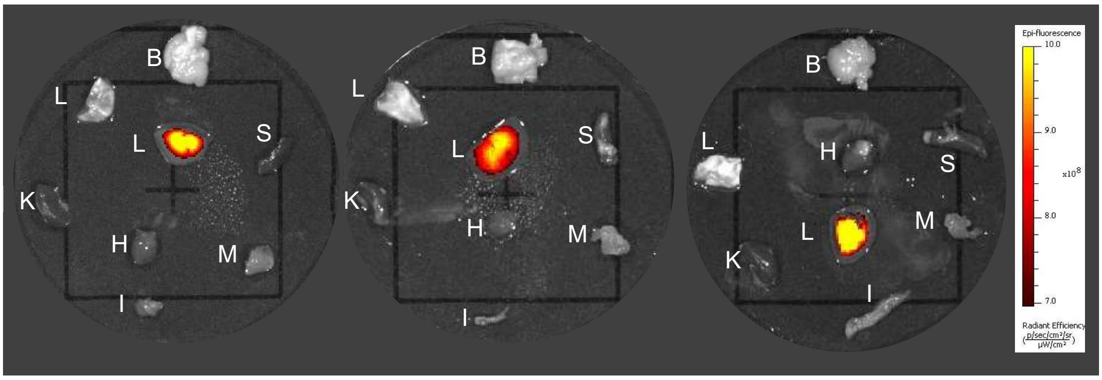

Figure S26. Representative images of mouse organs 24 h post injection of nanorods record using IVIS fluorescence imager. Left) L_n = 69 nm, Centre) L_n = 146 nm, Right) L_n = 284 nm. (B = brain, Lu = lungs, Li = liver, S = spleen, K = kidney, H = heart, M = muscle, I = intestine).

References

[1] K. Kempe, M. Lobert, R. Hoogenboom, U. S. Schubert, *J. Comb. Chem.* **2009**, *11*, 274–280.

[2] A. C. G. Weiss, H. G. Kelly, M. Faria, Q. A. Besford, A. K. Wheatley, C. S. Ang, E. J. Crampin, F. Caruso, S. J. Kent, *ACS Nano* **2019**, *13*, 4980–4991.
